# Rapid metabolite response in leaf blade and petiole as a marker for shade avoidance syndrome

**DOI:** 10.1186/s13007-020-00688-0

**Published:** 2020-10-27

**Authors:** Benny Jian Rong Sng, Gajendra Pratap Singh, Kien Van Vu, Nam-Hai Chua, Rajeev J. Ram, In-Cheol Jang

**Affiliations:** 1 Temasek Life Sciences Laboratory, 1 Research Link, National University of Singapore, Singapore, 117604 Singapore; 2 Department of Biological Sciences, National University of Singapore, Singapore, 117543 Singapore; 3 Disruptive & Sustainable Technologies for Agricultural Precision, 1 CREATE way, Singapore-MIT Alliance for Research and Technology, Singapore, 138602 Singapore; 4 Research Laboratory of Electronics, Massachusetts Institute of Technology, Cambridge, MA 02139 USA

**Keywords:** Shade avoidance syndrome, Raman spectroscopy, Phytochrome signaling, Biomarker, Early diagnosis, Leaf blade, Petiole

## Abstract

**Background:**

Shade avoidance syndrome (SAS) commonly occurs in plants experiencing vegetative shade, causing morphological and physiological changes that are detrimental to plant health and consequently crop yield. As the effects of SAS on plants are irreversible, early detection of SAS in plants is critical for sustainable agriculture. However, conventional methods to assess SAS are restricted to observing for morphological changes and checking the expression of shade-induced genes after homogenization of plant tissues, which makes it difficult to detect SAS early.

**Results:**

Using the model plant *Arabidopsis thaliana*, we introduced the use of Raman spectroscopy to measure shade-induced changes of metabolites in vivo. Raman spectroscopy detected a decrease in carotenoid contents in leaf blades and petioles of plants with SAS, which were induced by low Red:Far-red light ratio or high density conditions. Moreover, by measuring the carotenoid Raman peaks, we were able to show that the reduction in carotenoid content under shade was mediated by phytochrome signaling. Carotenoid Raman peaks showed more remarkable response to SAS in petioles than leaf blades of plants, which greatly corresponded to their morphological response under shade or high plant density. Most importantly, carotenoid content decreased shortly after shade induction but before the occurrence of visible morphological changes. We demonstrated this finding to be similar in other plant species. Comprehensive testing of *Brassica* vegetables showed that carotenoid content decreased during SAS, in both shade and high density conditions. Likewise, carotenoid content responded quickly to shade, in a manner similar to *Arabidopsis* plants.

**Conclusions:**

In various plant species tested in this study, quantification of carotenoid Raman peaks correlate to the severity of SAS. Moreover, short-term exposure to shade can induce the carotenoid Raman peaks to decrease. These findings highlight the carotenoid Raman peaks as a biomarker for early diagnosis of SAS in plants.

## Background

Plants are sessile organisms that are unable to escape from their environment even when it becomes unfavorable. As such, it is crucial for plants to sense their surroundings and mount appropriate responses to different stresses. In vegetative shade, poor light quantity (fluence) and quality (wavelength) affects photosynthesis and plant development [1]. Specifically, vegetative shade has reduced Red:Far-red (R:FR) light ratio, which is sensed by phytochromes, a family of R/FR-absorbing photoreceptors in plants [1, 2]. Upon exposure to vegetative shade, plants respond by reaching for more light to overcome the shaded condition. This adaptive response, known as shade avoidance syndrome (SAS), includes stem and petiole elongation, hyponastic leaves, reduced leaf development, early flowering, and increased senescence [1, 2]. However, SAS also weakens the plant’s structure and immunity [1, 2]. Hence, SAS affects many agronomic traits such as reduced grain yield or plant biomass [3, 4].

Studies of SAS have largely focused at the genetic level. Using the model plant *Arabidopsis thaliana*, many genes involved in the signaling mechanisms underlying SAS have been characterized [5–7]. While gene expression is important to understand the initial response of shade-avoiding plants, downstream changes in hormone and metabolite levels are also crucial in determining a plant’s final morphological and physiological response [8, 9].

Hormonal studies of SAS have become more comprehensive in recent years [8, 9], but so far, few studies have investigated changes in metabolites during SAS [10, 11]. Current analytical techniques for plant hormones/metabolites have improved with the technological advancement of chromatography and mass spectrometry [12, 13]. Although these conventional methods are specific and accurate, their analyses depend on the extraction method and metabolites of interest [14]. Moreover, sample preparation is usually a limiting factor in high-throughput plant hormone/metabolite analysis. Therefore, there is a need for new technologies to easily monitor metabolite levels in a non-invasive manner and in real-time.

In the last decade, optical spectroscopies, in particular Raman spectroscopy, have been widely used for real-time measurements of metabolites in microbial and mammalian cells [15–19]. Raman spectroscopy, discovered in 1928 by C.V. Raman and K.S. Krishnan [20], measures the inelastic scattering of laser light, which results in a characteristic ‘fingerprint’ of vibrational frequencies for each molecular species present. The convergence of interest in plant metabolomics and maturity of Raman spectroscopy have stimulated recent exploration of optical biomarkers for plant stress. In particular, carotenoid and anthocyanin Raman spectra were shown to have similar response in cold, high light, drought, and salinity stresses [21]. Despite its potential, further research using Raman spectroscopy in plants remains largely unexplored.

Here, using Raman spectroscopy with near-infrared (830 nm) excitation wavelength, we could detect metabolite changes owing to SAS in different plant tissues. Particularly, carotenoid levels in both leaf blades and petioles were highly responsive to shade conditions and its response was more dramatic in petioles. Carotenoid levels quickly decreased upon shade exposure and occurred prior to any visible changes in plant morphology, which allowed for real-time monitoring and early diagnosis of SAS. Importantly, this finding was consistent across multiple plant species that we examined, highlighting its suitability as a biomarker for SAS in plants.

## Results

### Raman spectra analysis of Arabidopsis leaf blades and petioles during SAS

**Fig. 1:**
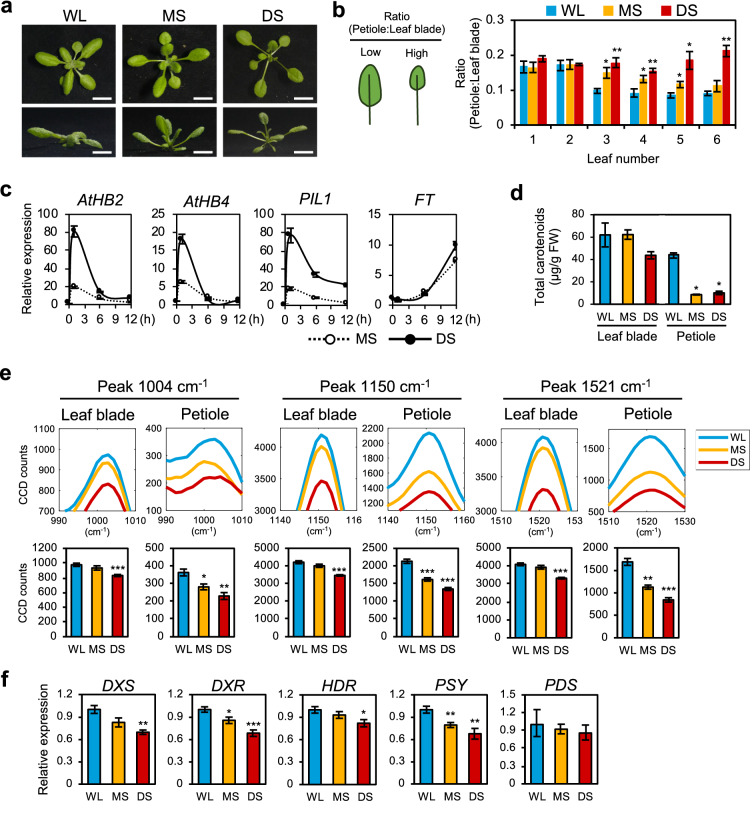
Application of Raman spectroscopy for SAS in Arabidopsis. **a** Phenotype of 17-days-old WT Arabidopsis plants under white light (WL) and two different shade conditions, moderate shade (MS) and deep shade (DS). Upper panel, top-down view of plant. Lower panel, side view of plant. Scale, 1 cm. **b** Ratio of petiole length to leaf blade area for visualising severity of SAS (n = 8). **c** Relative expression of shade-induced genes in Arabidopsis plants in **a** (n = 3). *AtHB2/4*, *A. thaliana Homeobox Protein 2/4*; *PIL1*, *Phytochrome Interacting Factor 3-Like 1*; *FT*, *Flowering Locus T*. **d** Total carotenoid content (µg/g fresh weight) of plants in **a** (n = 3). **e** Raman spectrum peaks representing carotenoids (1004 cm^−1^, 1150 cm^−1^ and 1521 cm^−1^) of Arabidopsis under shade conditions in **a** (leaf blade: n = 8, petiole: n = 4). **f** Relative gene expression of carotenoid biosynthesis genes in Arabidopsis plants in **a** (n = 3). *DXS*, *1-deoxy-D-xylulose-5-phosphate synthase*; *DXR*, *1-deoxy-D-xylulose 5-phosphate reductoisomerase*; *HDR*, *hydroxymethylbutenyl 4-diphosphate reductase*; *PSY*, *Phytoene Synthase*; *PDS*, *Phytoene Desaturase*. Bars denote average ± SE. Statistical significance between WL and shade was determined by two-tailed Student’s *t*-test: *P < 0.05; **P < 0.01; ***P < 0.001

To investigate if Raman spectroscopy could identify metabolites that change in response to shade, we established shade conditions that were low in Red:Far-red (R:FR) light to induce SAS in Arabidopsis plants. Wild-type (WT, Col-0) Arabidopsis seedlings (10 days after germination, DAG) were grown under three different light conditions: white light (WL) for normal growth of Arabidopsis, moderate shade (MS) for partial vegetative shade, and deep shade (DS) for severe shade for 7 days (d). Figure 1a, b and Additional file 1: Fig. S1a show that MS condition reproducibly induced typical SAS in WT, including hyponasty of rosette leaves, elongation of petioles and reduction of leaf blades. SAS became more obvious in DS condition, showing that our shade setup can induce different severities of SAS in plants (Fig. 1a, b, Additional file 1: Fig. S1a). These morphological changes were accompanied by the shade-induced expression of marker genes such as *A. thaliana Homeobox Protein 2/4* (*AtHB2/AtHB4*) and *Phytochrome Interacting Factor 3-Like 1* (*PIL1*), which were highly induced under short durations of MS and DS treatment (Fig. 1c) [7, 22]. Likewise, the induction of the flowering time gene *Flowering Locus T* (*FT*) in shaded conditions indicated the early flowering of SAS plants (Fig. 1c) [23].

We built a tabletop Raman spectroscopy instrument with specific near-infrared (830 nm) excitation wavelength. As light signaling in plants involves perception and response to visible light, we chose the 830 nm excitation laser to avoid activating any light signaling pathways. Moreover, this wavelength of light was found to provide the largest signal-to-background of the various excitation wavelengths considered (450–830 nm). The optical background here was dominated by off-resonant chlorophyll autofluorescence excited by the infrared laser. This infrared excitation wavelength falls within a spectral window of very low optical absorption in most plant leaves [24], resulting in negligible photodamage to the plant tissues or metabolites even if the laser exposure used is 30 times longer compared to that used here.

Using this purpose-built Raman spectroscopy system (Additional file 1: Fig. S1b), we investigated changes of metabolites in each rosette leaf of WT Arabidopsis. Basically, the spectral intensities were low in the old leaves and increased in accordance to their leaf number (Additional file 1: Fig. S1c). This suggests that the metabolites in the leaf became less concentrated as the leaf aged. During the aforementioned shade treatment, 10-days-old Arabidopsis plants were subjected to shade treatment for 7 days, which exposed the third rosette leaf to the full duration of shade treatment during its development (Additional file 1: Fig. S1d). Hence, to ensure that the sampled leaf accurately represented the effect of the shade treatment, the third rosette leaf was chosen for all further Raman spectroscopy measurements.

The spectra obtained for Arabidopsis leaf blades and petioles under shade treatments showed the same spectral pattern and peak numbers (Additional file 1: Fig. S2a). We could not find any additional or missing peaks within the spectral range measured by our current Raman system. However, we noted that the intensity of most peaks above 700 cm^−1^ Raman shift decreased under shade conditions. This result confirms previous studies, which showed that the levels of multiple metabolites decreased under shade [10, 11].

To identify the peaks with the largest change under shade conditions, we performed a principal component analysis (PCA) plot using these data (Additional file 1: Fig. S2b). As an additional verification of metabolite changes under shade, the clustering of points were clearly separated in WL and DS, while clustering of points obtained under MS was an intermediate between WL and DS. By plotting the PCA coefficients against Raman shift, we then identified the peaks at 1150 cm^−1^ and 1521 cm^−1^ Raman shift as the largest change under shade (PC1) (Additional file 1: Fig. S2b). Based on our Raman spectrum library of chemical standards, we identified the 1150 cm^−1^ and 1521 cm^−1^ peaks to be present in all tested carotenoids (Additional file 1: Fig. S2c). Furthermore, the carotenoid standards share a third Raman peak at 1004 cm^−1^ Raman shift (Additional file 1: Fig. S2c). A previous study showed that shade condition reduces carotenoid biosynthesis and the total carotenoid content in Arabidopsis [25], and our results here support these previous observations. For verification, we extracted and quantified total carotenoid content in our samples using conventional UV–VIS spectroscopy and found similar results (Fig. 1d).

Figure 1e shows that in Arabidopsis plants, the carotenoid Raman peaks (1004 cm^−1^, 1150 cm^−1^ and 1521 cm^−1^) highly decreased in DS in both leaf blade and petiole. All peaks have similar decreases in intensity: in leaf blades, the 1004 cm^−1^ peak decreased by 4% in MS and 15% in DS, the 1150 cm^−1^ peak decreased by 5% in MS and 18% in DS, and the 1521 cm^−1^ peak decreased by 4% in MS and 19% in DS (Fig. 1e). Interestingly, shade caused a greater decrease in peak intensity for carotenoid and other peaks in the petioles than in leaf blades (Fig. 1e, Additional file 1: Fig. S2a), as the 1004 cm^−1^ peak decreased 22% (MS) and 37% (DS), the 1150 cm^−1^ peak decreased by 24% (MS) and 37% (DS), and the 1521 cm^−1^ peak decreased by 33% (MS) and 50% (DS), suggesting that more metabolomic changes occurred in the petioles during SAS (Fig. 1e). As all peaks showed a similar trend under shade and the 1521 cm^−1^ peak was less affected by autofluorescence, subsequent experiments focused on the 1521 cm^−1^ peak as a representative of carotenoid Raman peaks.

To further verify the decreased carotenoid content in shade conditions, we measured the expression of genes related to carotenoid biosynthesis, which are known to be down-regulated in etiolated plants [26]. Expression levels of upstream methylerythritol 4-phosphate (MEP) pathway genes (*1-deoxy-D-xylulose-5-phosphate synthase*, *DXS*; *1-deoxy-D-xylulose 5-phosphate reductoisomerase*, *DXR*; *hydroxymethylbutenyl 4-diphosphate reductase*, *HDR*) and the first committed step of carotenoid biosynthesis (*Phytoene Synthase*, *PSY*) were down-regulated under shade conditions, with lower gene expression in DS (Fig. 1f). As such, these results show that carotenoid content decreased in both Arabidopsis leaf blades and petioles during SAS and were relative to the severity of the shade condition. Using Raman spectroscopy, we showed that carotenoid content in petioles were more responsive to shade than leaf blades.

### Early diagnosis of SAS using Raman spectroscopy

**Fig. 2:**
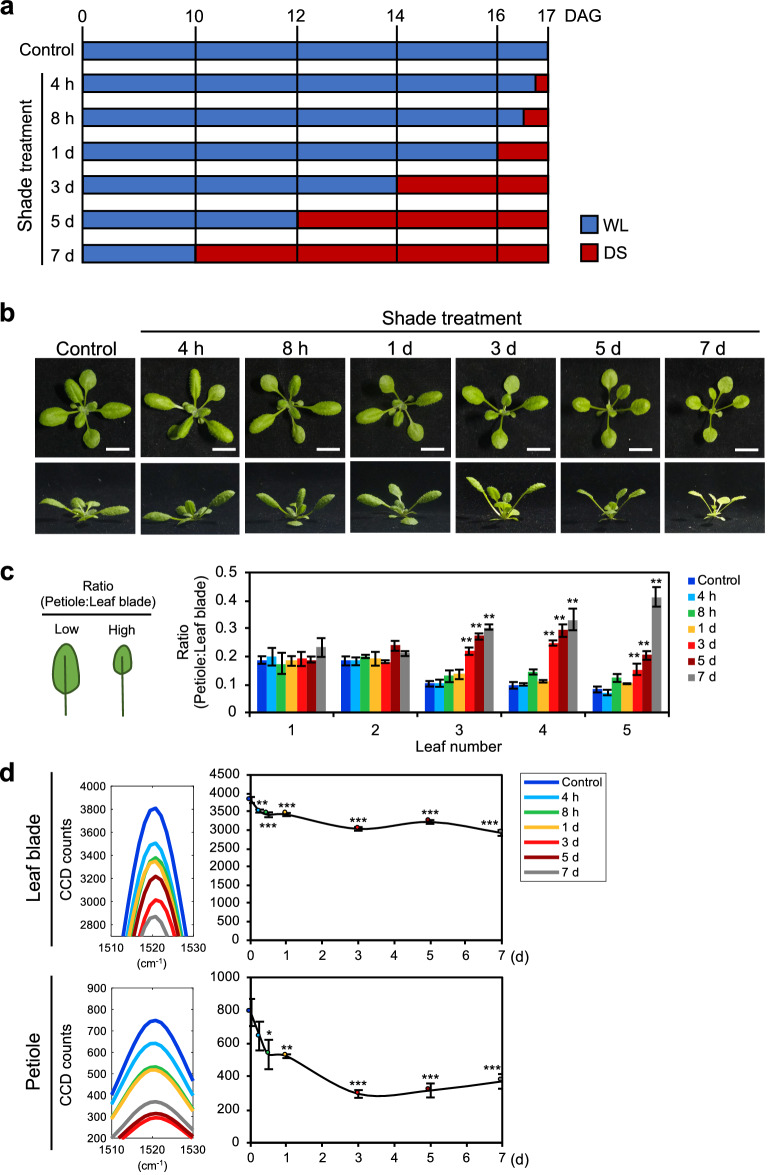
Time-course experiment of SAS using Raman spectroscopy in Arabidopsis. **a** Schematic diagram of different durations of shade treatment. WL, white light; DS, deep shade. **b** Phenotype of plants under time-course shade treatment. 10-days-old Arabidopsis plants were subjected to a time-course 7 days shade treatment as in **a**. Upper panel, top-down view of plant. Lower panel, side view of plant. Scale, 1 cm. **c** Ratio of petiole length to leaf blade area in time-course plants (n = 3). **d** Carotenoid Raman peak of time-course shade treatment in Arabidopsis plants in **b** (leaf blade: n = 10, petiole: n = 8). Bars and graph denote average ± SE. Statistical significance between control and different durations of shade treatment was determined by two-tailed Student’s *t*-test: *P < 0.05; **P < 0.01; ***P < 0.001

After establishing that the carotenoid Raman peaks are indicative of SAS, we then asked if these metabolites would respond early to shade. To test this, WT Arabidopsis plants were subjected to different durations of DS treatment before measuring their Raman spectra (Fig. 2a). With longer exposure to shade, the plants developed more severe SAS, with morphological changes after 1–3 days of shade treatment (Fig. 2b, c). Furthermore, morphological changes affected leaf number 3 onwards, verifying the measurement of the third rosette leaf in Raman spectroscopy (Fig. 2c). Surprisingly, changes in the carotenoid peak intensities were detected before morphological changes occurred, starting from just 4 h of DS condition (Figs. 2d, Additional file 1: Fig. S3). Carotenoid peak in both leaf blades and petioles decreased between 4 h to 3 days of shade treatment, reaching a steady state level from 3 days onwards (Fig. 2d). Similar to the results thus far, there was a larger decrease in peak intensity in the petioles (18% at 4 h to 53% at 7 days) compared to the leaf blades (8% at 4 h to 24% at 7 days), confirming that petioles were more reactive to shade. To account for ontogenetic effects on carotenoid content, we designed another time-course experiment, which tracked the daily development of SAS and Raman peak changes under WL and DS (Additional file 1: Fig. S4a). Similar results verified that prolonged shade treatment caused a large decrease in the carotenoid peak intensities, especially in the petioles (Additional file 1: Fig. S4b). Together, these results highlight that the intensity of the carotenoid peaks responded quickly to shade and occurred before obvious morphological changes. These results suggest that carotenoid Raman peaks can be used as a marker for early diagnosis of SAS.

### Reduced carotenoid content in phytochrome mutants

SAS is mediated by phytochrome signaling, which has photo-reversible activation/inactivation dependent on the R:FR ratio [27]. Among the five Arabidopsis phytochromes (PHYA-PHYE), PHYA and PHYB are regarded as key players in regulating SAS [27]. Under high R:FR PHYB is the predominant phytochrome that prevents SAS such as petiole elongation and reduction of leaf blade area, whereas under low R:FR PHYA blocks the excessive elongation of seedlings [27, 28].

**Fig. 3:**
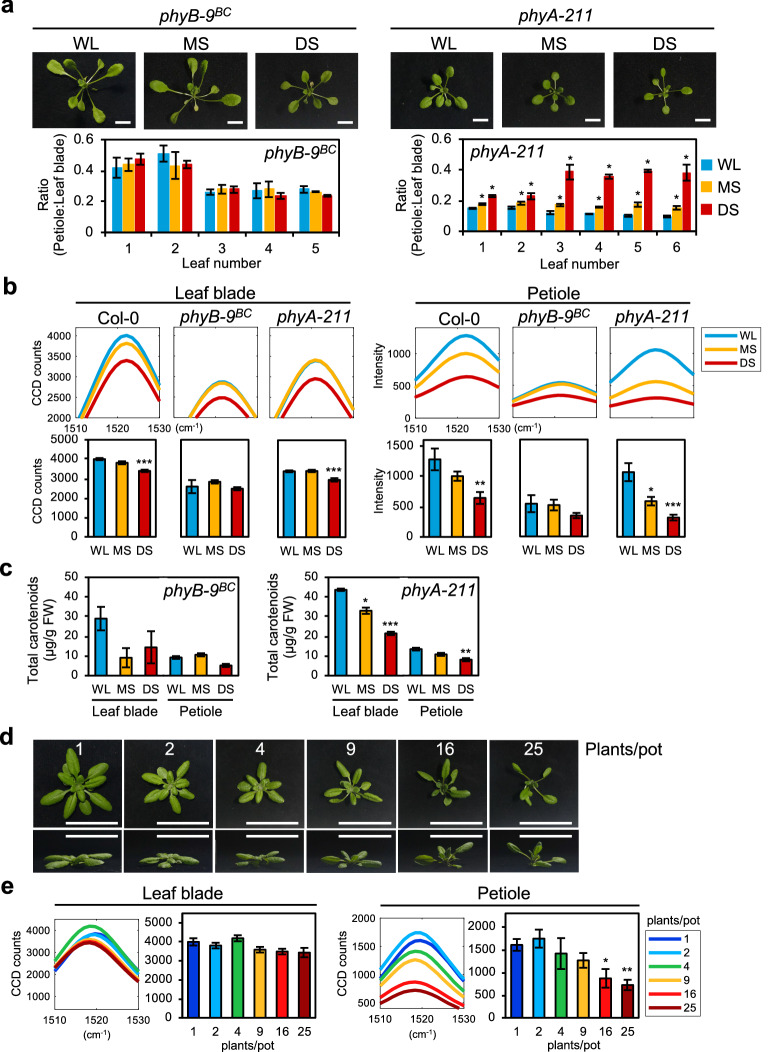
Raman spectra analysis of WT Arabidopsis and phytochrome mutants, and of WT Arabidopsis at high density planting. **a** Phenotype of *phyB-9*^*BC*^ and *phyA-211* after 7 days shade treatment and respective ratios of petiole length to leaf blade area. **b** Carotenoid Raman peak of plants in **a** (leaf blade: n = 8, petiole: n = 4). **c** Total carotenoid content (µg/g fresh weight) of plants in **a** (n = 3). **d** Phenotype of 24-days-old WT Arabidopsis plants grown in low to high plant density. Numbers represent plants per pot. Upper panel, top-down view of individual plant  from each pot. Lower panel, side view of  individual plant from each pot. Scale, 5 cm. **e** Carotenoid Raman peak of Arabidopsis under low to high plant density in **d** (leaf blade: n = 5, petiole: n = 3). Bars denote average ± SE. WL, white light; MS, moderate shade; DS, deep shade. Statistical significance between control (WL or 1 plant/pot) and the respective treatment (shade or high plant density) was determined by two-tailed Student’s *t*-test: *P < 0.05; **P < 0.01; ***P < 0.001

To investigate if the decreased intensity of carotenoid Raman peaks during SAS is associated with phytochrome signaling, we used Arabidopsis phytochrome mutants (*phyB-9*^*BC*^ and *phyA-211*) and measured their Raman spectra under shade conditions. Consistent with previous studies, *phyB-9*^*BC*^ displayed constitutive SAS, whereas *phyA-211* showed no SAS under WL but more severe SAS than WT when under shade (Fig. 3a, Additional file 1: Fig. S5a) [1, 28–30]. Under all growth conditions, *phyB-9*^*BC*^ displayed similar and very low intensities for carotenoid Raman peaks, reflecting its constitutive SAS (Figs. 3b, Additional file 1: Fig. S5b). In *phyA-211*, the carotenoid peaks in leaf blades and petioles decreased in MS and DS, following a similar trend as WT in shade (Fig. 3b, Additional file 1: Fig. S5b). However, *phyA-211* has lower intensities than WT under all conditions and *phyA-211* petioles have a larger decrease in intensity (45% in MS, 70% in DS) than WT petioles (22% in MS, 50% in DS) (Fig. 3b). This is consistent with the knowledge that PHYA senses low R:FR light and reduces the severity of SAS, thus the loss of PHYA causes a greater sensitivity to SAS [28]. Extraction of total carotenoids further verified the changes observed in the carotenoid Raman peaks (Fig. 3c). Therefore, the results show that the reduction in carotenoid content is caused by phytochrome-mediated SAS.

### Raman spectra analysis at high density planting

Besides vegetative shade, high density planting also causes low R:FR light and induces SAS [31]. To further verify our results obtained from the shade experiment, we planted Arabidopsis plants under low to high densities and measured their Raman spectra. Figure 3d shows that high plant densities (9–25 plants/pot) resulted in increasingly severe SAS in Arabidopsis plants. The measured Raman spectra reflected this change in SAS, as the carotenoid Raman peak had lower peak intensity at higher planting densities (Fig. 3e, Additional file 1: Fig. S6). Similar to the shade treatment results, the peak intensities in the petioles showed more significant decrease (55% decrease in 25 plants/pot) than those in the leaf blades (14% decrease in 25 plants/pot) (Fig. 3e). These results show that the carotenoid peak in a Raman spectrum can also be used as a marker to detect SAS caused by high density planting conditions in Arabidopsis.

### Raman spectra analysis of leafy vegetables under shade conditions

**Fig. 4:**
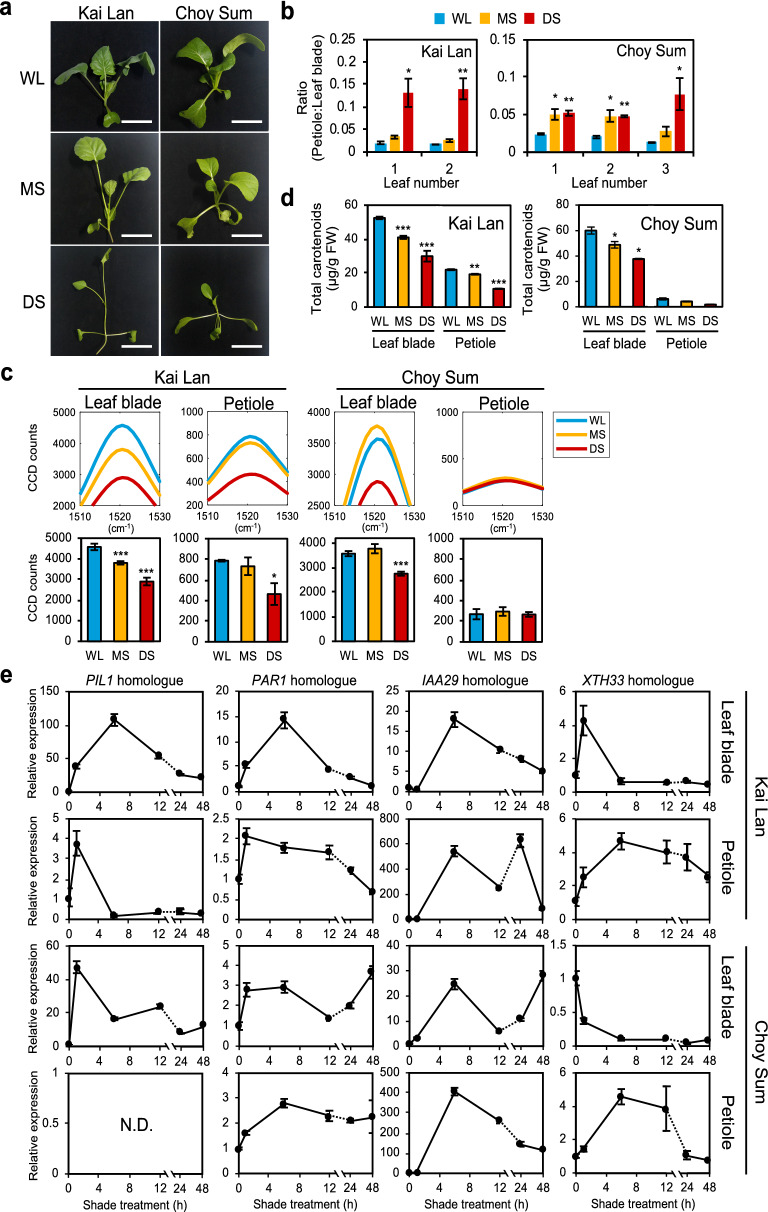
Raman spectra analysis of leafy vegetables under shade. **a** Phenotype of Kai Lan and Choy Sum in shade conditions. Scale, 5 cm. **b** Ratio of petiole length to leaf blade area of plants in **a** (n = 3). **c** Carotenoid Raman peak of Kai Lan and Choy Sum (Leaf blade: n = 8, petiole: n = 4). Bars denote average ± SE. **d** Total carotenoid content (µg/g fresh weight) from Kai Lan and Choy Sum in **a**. **e** Relative expression of homologues of Arabidopsis shade-induced genes in Kai Lan and Choy Sum leaf blades and petioles. X-axis represents duration of exposure to DS (n = 3). *PIL1*, *Phytochrome Interacting Factor 3-Like 1*; *PAR1*, *Phytochrome Rapidly Regulated 1*; *IAA29*, *Indole-3-acetic acid Inducible 29*; *XTH33*, *Xyloglucan:xyloglucosyl Transferase 33*. WL, white light; MS, moderate shade; DS, deep shade. N.D., not detected. Statistical significance between WL and shade was determined by two-tailed Student’s *t*-test: *P < 0.05; ***P < 0.001

To further validate the use of Raman spectroscopy in SAS, we investigated its application in *Brassica* species. Two species of leafy vegetables, Kai Lan (*Brassica oleracea* var. *alboglabra*) and Choy Sum (*Brassica rapa* var. *parachinensis*) were treated under shade for 14 days. While Kai Lan reacted to the shade conditions with both reduction of leaf blades and elongation of petioles, Choy Sum under shade developed smaller leaf blades but no significant elongation of the petioles (Fig. 4a, b, Additional file 1: Fig. S7b).

Raman spectroscopy was applied to the first true leaf of each vegetable as it received the full duration of shade treatment (Additional file 1: Fig. S7a). Figure 4c shows that the carotenoid peak of Kai Lan leaf blade and petiole decreased under both MS and DS (leaf blades, 17% and 37%; petioles, 7% and 41%, respectively). While Choy Sum leaf blades decreased their carotenoid peak intensity by 23% in DS, its petioles had no significant change regardless of the shade condition (Fig. 4c). This result may explain the lack of petiole elongation in Choy Sum compared to Kai Lan, especially in the true leaves (Fig. 4a, b, Additional file 1: Fig. S7b). The reduction in extracted total carotenoids in Kai Lan and Choy Sum under shade was verified by measuring total carotenoid content in homogenized plant tissues (Fig. 4d). Similar to Raman peak intensities, carotenoid content in petioles of Choy Sum was very low compared to that of Kai Lan (Fig. 4c, d). Together, these results show that SAS in Kai Lan is similar to Arabidopsis, whereas only Choy Sum leaf blades but not petioles responded to shade. Importantly, the differences between leaf blades and petioles can be clearly detected by measuring their respective carotenoid Raman peaks, making it a useful diagnostic tool for SAS in these *Brassica* species.

Interestingly, the 1045 cm^−1^ Raman peak in Kai Lan and Choy Sum petioles showed a distinctively opposite trend under shade. While most peaks in the Raman spectra decreased under shade conditions, the 1045 cm^−1^ Raman peak increased in only DS (Additional file 1: Fig. S7c). This suggests that Kai Lan and Choy Sum petioles specifically accumulate a different metabolite under DS, which was not observed in Arabidopsis.

To further investigate the difference between leaf blades and petioles of Kai Lan and Choy Sum during SAS, we measured the expression of homologues of Arabidopsis shade-induced marker genes (*PIL1*; *PAR1*, *Phytochrome Rapidly Regulated 1*; *IAA29*, *Indole-3-acetic acid Inducible 29*; *XTH33*, *Xyloglucan:xyloglucosyl Transferase 33*), which were up-regulated under shade treatment [32, 33]. Generally, gene expression fold-change in Kai Lan were either similar or higher than Choy Sum, which may explain the more severe SAS seen in Kai Lan (Fig. 4e). In both leaf blades and petioles of Kai Lan, the marker genes were highly up-regulated after short durations of shade treatment but gradually decreased over time (Fig. 4e). However, their expression patterns were slightly different in Choy Sum. For instance, the expression of *XTH33* homologue was down-regulated in Choy Sum leaf blades under shade and *PIL1* homologue was not detectable in Choy Sum petioles regardless of the shade duration (Fig. 4e). Moreover, while the expression of *PAR1* homologue in Kai Lan returned to the baseline over time, the *PAR1* homologues in Choy Sum (especially petioles) remained induced after prolonged shade treatment. In Arabidopsis, PAR1 inhibits phytochrome interacting factors (PIFs) from reducing carotenoid biosynthesis in shaded conditions [25]. Therefore, the prolonged induction of *PAR1* homologue in Choy Sum may explain the smaller decrease in carotenoid levels during SAS. These results may correlate the differences in SAS response and respective carotenoid Raman peaks between Kai Lan and Choy Sum petioles.

### Early diagnosis of SAS in leafy vegetables using Raman spectroscopy

**Fig. 5:**
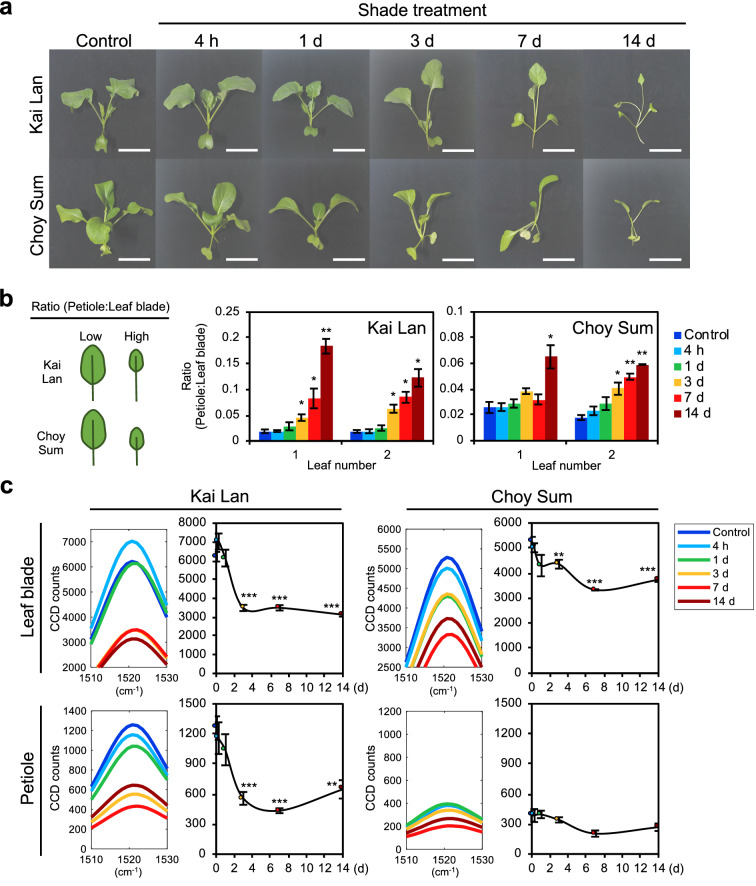
Time-course experiment of SAS using Raman spectroscopy in leafy vegetables. **a** Phenotype of leafy vegetables under time-course shade treatment. Basic strategy for shade treatment is the same as shown in Fig. 3a. 3- to 14-days-old Kai Lan and Choy Sum plants were grown in WL, followed by shade treatment in DS for 4 h to 14 days. All plants were of same age during measurement of phenotype and Raman spectroscopy. Scale, 5 cm. **b** Ratio of petiole length to leaf blade area of plants in **a** (n = 3). **c** Carotenoid Raman peaks of time-course shade treatment plants in **a** (n = 4). Bars and graph denote average ± SE. Statistical significance between control and different durations of shade treatment was determined by two-tailed Student’s *t*-test: *P < 0.05; **P < 0.01; ***P < 0.001

We then investigated if carotenoid Raman peaks can also be used in the early diagnosis of SAS in leafy vegetables as it was applicable in Arabidopsis. Both vegetables were subjected to a 14 days time-course experiment under DS condition, similar to the time-course shade experiment performed with Arabidopsis plants (Fig. 2a). Both Kai Lan and Choy Sum displayed increasingly severe SAS as the duration of shade treatment increased (Fig. 5a). Morphological changes in both Kai Lan and Choy Sum started after 3 days of shade treatment, as petioles of Kai Lan were elongated and leaf blades of both vegetables were reduced in size (Fig. 5b). Raman spectra of Kai Lan and Choy Sum in the time-course shade treatment showed that the carotenoid peak reduced in all samples during SAS except Choy Sum petioles (Fig. 5c, Additional file 1: Fig. S8), which is consistent with our findings in Fig. 4c. After 14 days of shade, the carotenoid peak of Kai Lan leaf blades and petioles decreased by 50% and 49% respectively, while Choy Sum leaf blades showed a 29% decrease at 14 days shade and Choy Sum petioles displayed no significant changes (Fig. 5c). Carotenoid peak intensity decreased within 1 to 3 days of shade treatment in Kai Lan and Choy Sum (Fig. 5c, Additional file 1: Fig. S8). This decrease preceded clear morphological changes of leafy vegetables under shade condition (Fig. 5b). Therefore, these results show the relative shade tolerance in Choy Sum and demonstrated that the decrease in carotenoid Raman peaks can be used in early identification of SAS in *Brassica* vegetables.

### Raman spectra analysis of leafy vegetables grown at high density

**Fig. 6:**
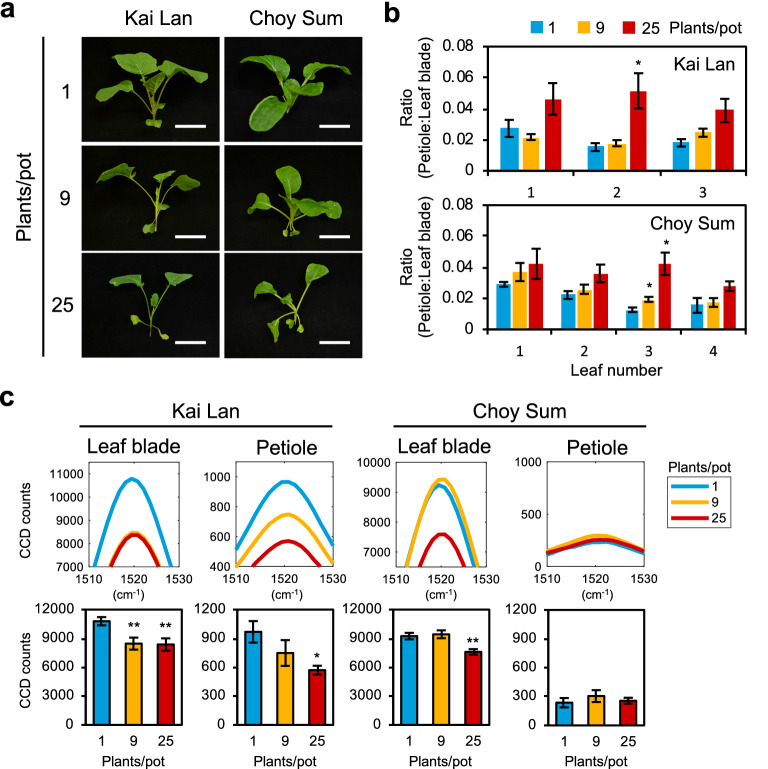
Raman spectra analysis of leafy vegetables at high density planting. **a** Phenotype of Kai Lan and Choy Sum in low to high density conditions (plants/pot). Scale, 5 cm. **b** Ratio of petiole length to leaf blade area of leafy vegetables in **a** (n = 3). **c** Carotenoid Raman peaks of Kai Lan and Choy Sum (leaf blade: n = 6, petiole: n = 4). Bars denote average ± SE. Statistical significance between control (1 plant/pot) and high density planting was determined by two-tailed Student’s *t*-test: *P < 0.05; **P < 0.01

Next, Raman spectra analysis was performed in Kai Lan and Choy Sum grown at low to high plant densities. At high density, both Kai Lan and Choy Sum developed SAS that were similar to those under shade conditions (Fig. 6a, b, Additional file 1: Fig. S9a). The carotenoid Raman peak also followed a similar trend to those observed in the shade conditions (Figs. 4c, 6c), thereby showing that this finding is likewise applicable in high density growing of non-Arabidopsis plants.

### Detection of SAS across various plant species using Raman spectroscopy

**Fig. 7:**
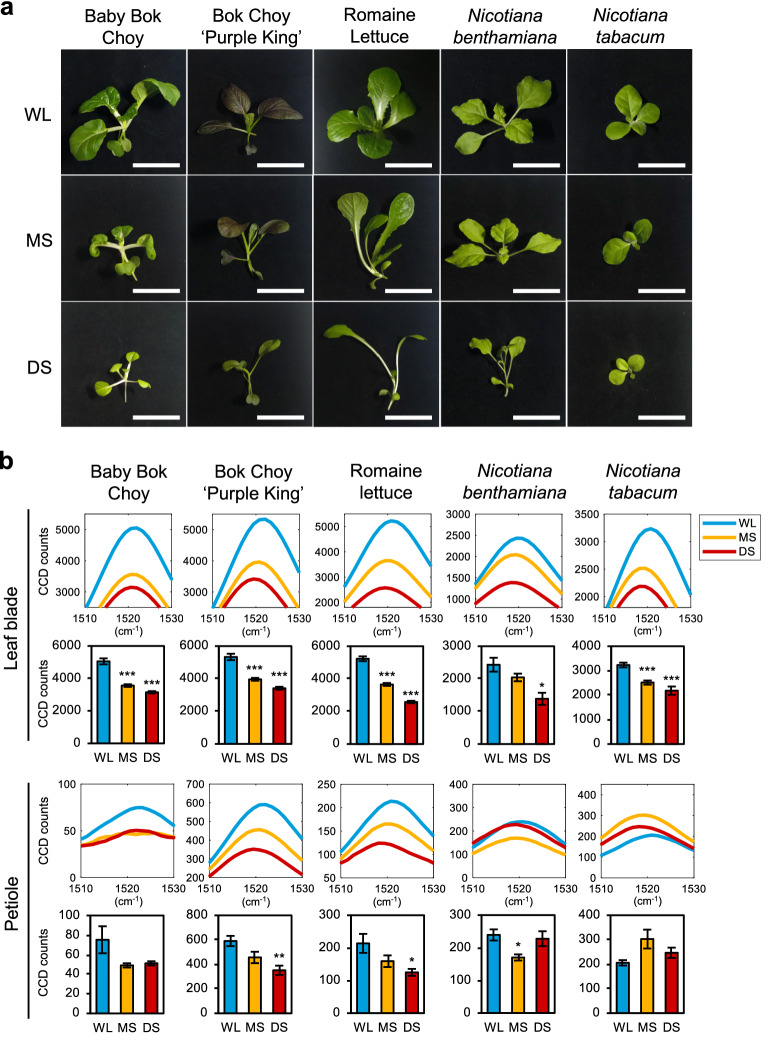
Raman spectra of various plant species under shade conditions. **a** Phenotype of various plant species grown in shade. Scale, 5 cm. **b** Carotenoid Raman peaks of leaf blade and petiole of plants in **a** (Baby Bok Choy: leaf blade n = 7, petiole n = 4; Bok Choy ‘Purple King’: leaf blade n = 6, petiole n = 6; Romaine Lettuce: leaf blade n = 6, petiole n = 6; *Nicotiana benthamiana*: leaf blade n = 3, petiole n = 3; *Nicotiana tabacum*: leaf blade n = 6, petiole n = 3). WL, white light; MS, moderate shade; DS, deep shade. Bars denote average ± SE. Statistical significance between WL and shade was determined by two-tailed Student’s *t*-test: *P < 0.05; **P < 0.01; ***P < 0.001

To demonstrate the general utility of Raman spectroscopy for the early diagnosis of SAS in plants, we tested other types of vegetables and plants, including Bok Choy cultivars (*Brassica rapa* var. *chinensis*), Romaine Lettuce (*Lactuca sativa* L. var. *longifolia*), and two tobacco species, *Nicotiana benthamiana* and *Nicotiana tabacum*. Figure 7a shows that all tested plants displayed different degrees of SAS when subjected to shade treatment. Baby Bok Choy, Bok Choy ‘Purple King’, and Romaine lettuce were more sensitive to shade compared to the two tobacco species (Fig. 7a). These were detected by Raman spectroscopy, as the carotenoid peak intensities decreased in both leaf blades and petioles of two Bok Choy cultivars and Romaine lettuce in MS and DS (Fig. 7b). The 1045 cm^−1^ Raman peak also clearly increased in Romaine lettuce petioles under shade (Additional file 1: Fig. S10), which is similar to Kai Lan and Choy Sum petioles in DS (Additional file 1: Fig. S7c). Plants of *N. benthamiana* showed clear SAS only in DS, while *N. tabacum* did not show any elongation of petioles under shade (Fig. 7a). This greater tolerance to shade corresponds to the smaller decrease in carotenoid peak intensities of leaf blades under shade and no clear trend in the petiole carotenoid peak (Fig. 7b). Overall, our results demonstrate that carotenoid levels and its Raman peaks are widely applicable as a biomarker of SAS regardless of the plant species.

## Discussion

In this study, we showed that the decrease of total carotenoids in plants, which is indicative of SAS, can be detected by Raman spectroscopy as three major peaks in the Raman spectra (1004 cm^−1^, 1150 cm^−1^ and 1521 cm^−1^). While a previous study showed the potential of Raman spectroscopy in identifying general abiotic stress in Coleus lime [21], here we used multiple plant species that showed different degrees of shade response and measured Raman spectra in different tissues (leaf blades and petioles). Additionally, we performed comprehensive experimental approaches for SAS study and found total carotenoids as a Raman spectroscopy biomarker for SAS. While a previous report showed that the total carotenoid content in Arabidopsis was reduced during SAS [25], the results here further demonstrated it in other plant species, including *Brassica* vegetables, lettuce, and tobacco plants. Likewise, the 1521 cm^−1^ carotenoid peak intensity decreased under shade for all plants with SAS. Although the 1521 cm^−1^ peak is known to overlap with Raman signals of anthocyanins [34], the changes to 1150 cm^−1^ and 1521 cm^−1^ carotenoid peaks were similar in all of our experiments, suggesting that our results were not affected by the overlap.

Our results suggest that the change in carotenoid content may be correlated with the development of typical SAS, such as shade-induced petiole elongation and inhibition of leaf blade expansion. This hypothesis is supported by our observation that Arabidopsis *phyB-9*^*BC*^ mutant, having constitutive SAS, showed very low carotenoid peak intensities that did not change in leaf blades and petioles regardless of shade. Similarly, petioles of Choy Sum and tobacco species, which did not significantly elongate under shade, showed minimal changes in carotenoid peak intensities. All other plant samples that showed SAS under shade displayed a corresponding decrease in carotenoid peak intensities. These changes were verified by quantification of extracted total carotenoids and the reduced expression of carotenoids biosynthesis genes in shade.

Moreover, we found the 1045 cm^−1^ Raman peak to increase in only petioles of Choy Sum, Kai Lan, and Romaine lettuce under shade. This Raman peak was recently identified to be a nitrate Raman peak in Arabidopsis and leafy vegetables [35], but further study is needed to investigate the reason for its induction in petioles of Choy Sum, Kai Lan, and Romain lettuce under shade.

As our Raman spectroscopy system allowed us to sample small areas of a leaf, we discovered that the changes in carotenoid peak intensities under shade were remarkably different between leaf blades and petioles, as well as between plant species. This suggests that while different plant species vary in their sensitivity to shade, shade-related genes also respond differently in leaf blades and petioles as shown in Fig. 4e, which ultimately results in different levels of metabolites accumulated during SAS. Separate analysis of leaf blade and petiole metabolite content using a non-invasive method has not been well-studied thus far. Hence, our finding in this study highlights the importance and applicability of Raman spectroscopy in analyzing the heterogeneity of metabolites within different parts of a plant, which can be applied to other studies besides SAS. For example, Raman spectroscopy was used to detect natural plant metabolites in the roots of gentian, turmeric, ginger, and *Triphyophyllum peltatum* [36, 37]. However, the use of Raman spectroscopy to study abiotic stress response in root tissues is still unexplored. Furthermore, future studies could explore the use of Raman spectroscopy to understand how plants regulate metabolites and for rapid screening of germplasms.

Conventional analytical methods in plant research using mass spectrometry-based techniques and nuclear magnetic resonance spectroscopy require homogenization of plant tissues, followed by extraction and quantification of the metabolite [14], which can be laborious and unsuitable for in vivo monitoring of metabolites. Moreover, as extracted metabolites have variable stability, even minor changes in procedure can have a major impact on the observed metabolome. Using a purpose-built Raman spectroscopy system, we were able to demonstrate carotenoid Raman peaks as a biomarker for early diagnosis and real-time monitoring of SAS in vivo and in a non-invasive manner with the following advantages: this method (1) takes only a minute to perform each measurement, allowing for faster and easier quantification per sample, (2) is not harmful to the plant as there is no pretreatment, (3) eliminates bias that may be introduced by sample preparation for conventional analysis [38], and (4) enables us to focus on individual seedlings, specific tissues (e.g. leaf blade or petiole) or cells. Reflectance spectroscopy and hyperspectral imaging are other rapid and non-invasive techniques that can provide information about the relative concentration of photopigments [18, 39–41]. Raman spectra of carotenoids exhibit narrow spectral features which allow us to clearly assess their abundance. Even so, certain carotenoid Raman peaks may overlap with the peaks from other compounds. To ensure that these measurements specifically represent the changes to total carotenoid content, all three carotenoid Raman peaks should be observed for proportional decrease in intensity. Unbiased analysis of the data using PCA shows that these peaks vary together–appear together as one principal component–indicating that they are vibrational states of the same molecule. Higher resolution Raman analysis can distinguish between peak intensities and positions of different carotenoids to measure changes to specific carotenoids, instead of inferring total carotenoids as a single peak. Finally, the data presented here uses the intensity of the carotenoid peak internally referenced to the Raman spectral peak intensity at 590 cm^−1^. Such an internal reference may not be readily available for other spectroscopic techniques making them sensitive to leaf morphology and orientation. Hence, our finding in this study highlights the importance and applicability of Raman spectroscopy in analyzing the heterogeneity of metabolites within different parts of a plant. We note that the Raman peak responses may differ between plant species and the duration of shade treatment. To address this, Raman spectra information of different plant species undergoing SAS can be stored in a database for users to assess their plant’s condition.

## Conclusions

Our study showcased the potential application of Raman spectroscopy in early diagnosis of SAS. Severity of SAS response can be quantified by the carotenoid Raman peak intensity. In addition, significant decrease in the peak intensity can be detected after short duration of shade treatment. By using phytochrome mutants, we verified this decrease in the carotenoid peak to be attributed to SAS. Decrease of carotenoid Raman peaks during shade were reproducible under natural growing conditions at high plant-density, which mimics the situation in commercial agriculture. By demonstrating this method across various plant species, we showed that this method is widely applicable for detecting SAS in plants.

## Methods

### Plant materials and plant growth

We used wild-type (Col-0), *phyB-9*^*BC*^, *phyA-211* as *A. thaliana* materials [30, 42]. All vegetable seeds were purchased from Ban Lee Huat Pte. Ltd. Singapore.

All seeds were subjected to cold stratification at 4 °C in darkness for 3 days before germination and growth in soil at 21 °C, 60% relative humidity under long day conditions (16 h light/8 h dark) and in WL (PPFD = 100 µmol cm^−2^ s^−1^, R:FR = 3.0). All seedlings except leafy vegetables were transplanted to individual pots 7 days after germination (DAG), and then subjected to their respective treatments. Leafy vegetable seedlings were transplanted 2 DAG, followed by their respective treatments.

### Shade and plant density treatments

Light treatment includes three conditions as follows: WL as a control (PPFD = 100 µmol cm^−2^ s^−1^, R:FR = 3.0), MS (PPFD = 60 µmol cm^−2^ s^−1^, R:FR = 0.7), and DS (PPFD = 30 µmol cm^−2^ s^−1^, R:FR = 0.2). WL was provided by Panasonic fluorescent tubes FL40SS and FR light was supplemented by CCS Asia ISL-150X150FR light emitting diodes (LEDs) to achieve the R:FR ratios. Top-down lighting was provided in all experiments.

*Arabidopsis thaliana* seedlings at 10 DAG were subjected to 7 days of shade treatment, followed by immediate phenotype measurements or Raman spectroscopy. Vegetable seedlings were grown to 3 DAG, followed by 14 days of shade treatment. *N. benthamiana* and *N. tabacum* seedlings were grown to 10 DAG and subjected to 14 days of shade treatment.

For the plant density experiment, all *A. thaliana*, Kai Lan and Choy Sum seedlings were germinated, grown, and transplanted as described above. Plants were transplanted into different densities and grown until 24 DAG under WL, followed by immediate phenotype measurement or Raman spectroscopy.

### Measurement of plant phenotype

Plants were dissected into individual leaves and, if applicable, its stem. Digital photographs of the dissected plant were taken. Petiole length, leaf blade area, and stem length were subsequently measured by using the photographs in ImageJ software [43] and LeafJ plugin [44].

### Raman spectroscopy

Raman spectra were measured using a purpose-built system designed for 830 nm excitation. The sample holder featured a 100 µm thick fused silica sampling window used for both excitation and collection of the Raman signal. An aspheric lens was used to focus the excitation light and collected the Raman scattered light. The lens was chosen with a depth of > 1 mm focus, so that the Raman signal from the entire cross-section of a leaf was collected. The excitation laser used with this system was a fiber coupled laser (Innovative Photonic Solutions, USA) operating at 830 nm, delivering approximately 100 mW of laser power to the sample. Light was delivered from the laser to collimating optics via a 105 micron core multimode fiber. The collimated light was passed through a Semrock MaxLine Laser Line 830 filter (Semrock Inc., USA) to remove any amplified spontaneous emission from the laser and any background generated within the delivery fiber. The filtered light was coupled into the optical path of the excitation lens by 830 nm RazorEdge Dichroic^™^ laser beamsplitter (Semrock Inc., USA) operated as a dichroic mirror. Collected light was passed back through the Semrock filter and then through additional 830 nm RazorEdge^®^ ultrasteep long-pass edge filter to further attenuate Rayleigh scattered excitation light before being delivered to the spectrometer using an F# matching lens. Spectra were acquired using Kymera 328i spectrograph (Andor, UK) employing the grating was 600 g/mm, blazed at 850 nm wavelength. For each sample of plant leaf, 5 spectra were collected with an integration time of 10 s per sample spot. Cosmic ray events were identified in the 10 s spectra and removed. After cosmic ray removal, the individual 10 s spectra were smoothed across wavelength using the Savitzky-Golay filter function (MATLAB Inc., USA) with 5th order polynomial and frame length of 11. A representative sample spectrum was created by taking the mean value of the five filtered and smoothed spectra at each wavelength. The sample spectrum resulting from this processing contained Raman and fluorescence signal primarily from the leaf. To generate the leaf Raman spectra presented in the results section, any residual fluorescence was removed by performing a positive residual style polynomial subtraction as described elsewhere [45]. Calibration of the Raman shift was performed using a polystyrene sample with a well-known Raman spectrum [46]. Raman spectra CCD counts were normalised to the 590 cm^−1^ Raman shift before comparison between samples.

### Plant samples for Raman spectroscopy

For *A. thaliana*, to ensure that the leaf received the full shade treatment, leaf blades and petioles of the third true leaf were used for measuring the Raman spectrum. Similarly, for Kai Lan and Choy Sum, leaf blades and petioles of the first true leaf were chosen for Raman spectroscopy.

We measured the Raman spectra from two locations per leaf blade (one on each side of the midvein) and one location in the middle of the petiole. A minimum of three biological replicates were used per plant sample. Two-tailed Student’s *t*-test was used to determine P-values.

### RNA extraction and quantitative reverse transcriptase-polymerase chain reaction (qRT-PCR)

Total RNA was extracted from finely ground plant samples using Ribospin Plant (GeneAll). The concentration of extracted RNA was determined by using NanoDrop 2000 Spectrophotometer (Thermo Fisher Scientific). Reverse transcription was performed using M-MLV reverse transcriptase (Promega).

Relative gene expression were quantified by 7900HT Fast Real-Time PCR system (Applied Biosystems). The reaction mixture consisted of TB Green^®^ Premix Ex Taq^™^ (Tli RNase H Plus) ROX Plus (TaKaRa), cDNA from plant samples, and primer pairs in Additional file 2: Table S1.

*A. thaliana* gene sequences were referred from the Arabidopsis Information Resource (https://www.arabidopsis.org), and gene expression was normalized to *UBQ11* as the internal control. Reference gene sequences for Choy Sum (Brassica rapa FPsc v1.3) and Kai Lan (Brassica oleracea capitata v1.0) were obtained from Phytozome [47]. Homologues with the highest similarity to Arabidopsis genes were selected and gene expression was normalized to homologues of Arabidopsis *ACT2*.

### Measurement of total carotenoid content by ultraviolet–visible (UV–VIS) spectrophotometer

Plant samples were frozen in liquid nitrogen and ground into a fine powder before extraction. Fresh weight was measured and used for normalization between samples. To extract total carotenoids, 100 mg fresh weight of the sample was resuspended in 1 mL of 100% methanol and kept on ice in darkness for 20 min. After centrifugation at 16000*g* at 4 °C for 4 min, the supernatant was transferred to a separate tube. Sample extractions were repeated until the sample loses all coloration. All extracts were pooled together. Absorbance values at 470 nm, 653 nm, and 666 nm were measured using Spark multimode microplate reader (Tecan) and total carotenoids were calculated based on the formula as described elsewhere [48].

## Supplementary information

**Additional file 1: Fig. S1.** Verification of shade conditions and determining leaf number of Arabidopsis plant for Raman spectroscopy. a Measurements of petiole length and leaf blade area of wild type (Col-0) Arabidopsis plants grown in shade conditions. Bars denote average ± SE (n = 4). b Left panel, schematic diagram of purpose-built Raman Spectroscopy system used in this study. Right panel, photograph of the Raman Spectroscopy system. Inset shows the leaf of a plant resting on the sample holder for measurement. c Left panel, Raman spectrum for each leaf number. Inset focused on 1521 cm^−1^ Raman peak. Right panel, measured peak intensities at 1521 cm^−1^ Raman shift. Bars denote average ± SE (n = 8). d Development of leaf number 3 of Arabidopsis plant. Numbers represent leaf number, according to order of development. C represents cotyledon. Scale, 1 cm. WL, white light; MS, moderate shade; DS, deep shade; DAG, days after germination.** Fig. S2.** Raman spectra of Arabidopsis plant under shade conditions. a Raman spectra of Arabidopsis leaf blades and petioles under different shade conditions. (leaf blade: n = 8, petiole: n = 4). b Three-dimensional principal component analysis (PCA) plot of wild type (Col-0) Arabidopsis under shade conditions. c Raman spectra for carotenoid standards. WL, white light; MS, moderate shade; DS, deep shade.** Fig. S3.** Raman spectra of same-age wild type (Col-0) Arabidopsis plants with different duration of shade treatment. Number of hours (h) or days (d) represents the duration of shade treatment. Control plants are not exposed to shade. (leaf blade: n = 10, petiole: n = 8).** Fig. S4.** Raman spectra of Arabidopsis plants from seedling to mature stage in time-course shade experiment. a Phenotype of plants at different age and different durations of shade treatment. 10-days-old plants were subjected to 7 days treatment of white light (WL) or deep shade (DS). b Carotenoids Raman peak of plants in a. Dashed line indicates difference in peak intensity between WL and DS. DAG, days after germination.** Fig. S5.** Raman spectra of Arabidopsis phytochrome mutants under shade conditions. a Petiole length and leaf blade area of *phyB-9*^*BC*^ and *phyA-211* under shade. Bars denote average ± SE (n = 3). b Raman spectra of leaf blades and petioles of *phyB-9*^*BC*^ and *phyA-211* in a (leaf blade: n = 8, petiole: n = 4). WL, white light; MS, moderate shade; DS, deep shade. **Fig. S6.** Raman spectra of wild type (Col-0) Arabidopsis leaf blades and petioles in low to high density planting. (leaf blade: n = 5, petiole: n = 3). **Fig. S7.** Raman spectra of leafy vegetables under shade conditions. a Development of leaf number 1 (blue arrowhead) in vegetables. Scale, 2 cm. b Measurements of petiole length, leaf blade area, hypocotyl length, and epicotyl length of Kai Lan and Choy Sum grown in shade conditions. Bars denote average ± SE (n = 3). c Raman spectra of leaf blades and petioles of Kai Lan and Choy Sum in a (leaf blade: n=8, petiole: n = 4). WL, white light; MS, moderate shade; DS, deep shade; C, cotyledon. **Fig. S8.** Raman spectra of leafy vegetables with different duration of shade treatment. a Raman spectra of leaf blades and petioles of Kai Lan (n = 5). b Raman spectra of leaf blades and petioles of Choy Sum (n = 4).** Fig. S9.** Raman spectra of leafy vegetables in high density planting. a Measurements of petiole length, leaf blade area, hypocotyl length, and epicotyl length of Kai Lan and Choy Sum grown in low to high density conditions. Bars denote average ± SE (n = 4). b Raman spectra of leaf blades and petioles of Kai Lan and Choy Sum in a (leaf blade: n = 6, petiole: n = 4). **Fig. S10.** Raman spectra of various plant species under shade conditions. (Baby Bok Choy: leaf blade n = 7, petiole n = 4; Bok Choy ‘Purple King’: leaf blade n = 6, petiole n = 6; Romaine Lettuce: leaf blade n = 6, petiole n = 6; Nicotiana benthamiana: leaf blade n = 3, petiole n = 3; Nicotiana tabacum: leaf blade n = 6, petiole n = 3). WL, white light; MS, moderate shade; DS, deep shade.

**Additional file 2: Table S1.** List of primers used in qRT-PCR analysis.

## Data Availability

All dataset supporting the conclusions of this article is included within the article and its Additional files 1 and 2.

## References

[1] Franklin KA, Whitelam GC (2005). Phytochromes and shade-avoidance responses in plants. Ann Bot.

[2] Ballaré CL, Pierik R (2017). The shade-avoidance syndrome: multiple signals and ecological consequences. Plant Cell Environ.

[3] Tang Y, Liesche J (2017). The molecular mechanism of shade avoidance in crops–How data from Arabidopsis can help to identify targets for increasing yield and biomass production. J Integr Agr.

[4] Wille W, Pipper CB, Rosenqvist E, Andersen SB, Weiner J (2017). Reducing shade avoidance responses in a cereal crop. AoB Plants..

[5] Casal JJ. Shade avoidance. In: The arabidopsis book, vol. 10, 2012. e0157. 10.1199/tab.0157 PMC3350169 22582029

[6] Devlin PF, Yanovsky MJ, Kay SA (2003). A genomic analysis of the shade avoidance response in Arabidopsis. Plant Physiol.

[7] Rolauffs S, Fackendahl P, Sahm J, Fiene G, Hoecker U (2012). Arabidopsis COP1 and SPA genes are essential for plant elongation but not for acceleration of flowering time in response to a low red light to far-red light ratio. Plant Physiol.

[8] Chaiwanon J, Wang W, Zhu JY, Oh E, Wang ZY (2016). Information integration and communication in plant growth regulation. Cell.

[9] Yang C, Li L (2017). Hormonal regulation in shade avoidance. Front Plant Sci.

[10] Caldana C, Degenkolbe T, Cuadros-Inostroza A, Klie S, Sulpice R, Leisse A, Steinhauser D, Fernie AR, Willmitzer L, Hannah MA (2011). High-density kinetic analysis of the metabolomic and transcriptomic response of Arabidopsis to eight environmental conditions. Plant J.

[11] Jänkänpää HJ, Mishra Y, Schröder WP, Jansson S (2012). Metabolic profiling reveals metabolic shifts in Arabidopsis plants grown under different light conditions. Plant Cell Environ.

[12] Kumar R, Bohra A, Pandey AK, Pandey MK, Kumar A (2017). Metabolomics for plant improvement: status and prospects. Front Plant Sci.

[13] Ren J, Zhang A, Kong L, Wang X (2018). Advances in mass spectrometry-based metabolomics for investigation of metabolites. RSC Adv.

[14] Jones WP, Kinghorn AD, Sarker S, Nahar L (2012). Extraction of plant secondary metabolites. Methods in Molecular Biology (Methods and Protocols).

[15] Chen N, Rong M, Shao X, Zhang H, Liu S, Dong B, Xue W, Wang T, Li T, Pan J (2017). Surface-enhanced Raman spectroscopy of serum accurately detects prostate cancer in patients with prostate-specific antigen levels of 4–10 ng/mL. Int J Nanomed.

[16] Ding J, Xu T, Tan X, Jin H, Shao J, Li H (2017). Raman spectrum: A potential biomarker for embryo assessment during in vitro fertilization. Exp Ther Med.

[17] Ember KJI, Hoeve MA, McAughtrie SL, Bergholt MS, Dwyer BJ, Stevens MM, Faulds K, Forbes SJ, Campbell CJ (2017). Raman spectroscopy and regenerative medicine: a review. NPJ Regen Med.

[18] Lorenz B, Wichmann C, Stöckel S, Rösch P, Popp J (2017). Cultivation-free Raman spectroscopic investigations of bacteria. Trends Microbiol.

[19] Shalabaeva V, Lovato L, La Rocca R, Messina GC, Dipalo M, Miele E, Perrone M, Gentile F, De Angelis F (2017). Time resolved and label free monitoring of extracellular metabolites by surface enhanced Raman spectroscopy. PLoS ONE.

[20] Raman CV, Krishnan KS (1928). A new type of secondary radiation. Nature.

[21] Altangerel N, Ariunbold GO, Gorman C, Alkahtani MH, Borrego EJ, Bohlmeyer D, Hemmer P, Kolomiets MV, Yuan JS, Scully MO (2017). In vivo diagnostics of early abiotic plant stress response via Raman spectroscopy. Proc Natl Acad Sci USA.

[22] Leivar P, Monte E, Cohn MM, Quail PH (2012). Phytochrome signaling in green Arabidopsis seedlings: impact assessment of a mutually negative phyB-PIF feedback loop. Mol Plant.

[23] Schwartz CJ, Lee J, Amasino R (2017). Variation in shade-induced flowering in Arabidopsis thaliana results from FLOWERING LOCUS T allelic variation. PLoS ONE.

[24] Merzlyak MN, Chivkunova OB, Melø TB, Naqvi KR (2002). Does a leaf absorb radiation in the near infrared (780–900 nm) region? A new approach to quantifying optical reflection, absorption and transmission of leaves. Photosynth Res.

[25] Bou-Torrent J, Toledo-Ortiz G, Ortiz-Alcaide M, Cifuentes-Esquivel N, Halliday KJ, Martinez-García JF, Rodriguez-Concepcion M (2015). Regulation of carotenoid biosynthesis by shade relies on specific subsets of antagonistic transcription factors and cofactors. Plant Physiol.

[26] Ruiz-Sola MÁ, Rodríguez-Concepción M. Carotenoid biosynthesis in Arabidopsis: a colorful pathway. In: The arabidopsis book, vol. 10, 2012, e0158. 10.1199/tab.0158 PMC3350171 22582030

[27] Franklin KA, Quail PH (2010). Phytochrome functions in Arabidopsis development. J Exp Bot.

[28] Yang C, Xie F, Jiang Y, Li Z, Huang X, Li L (2018). Phytochrome A negatively regulates the shade avoidance response by increasing Auxin/Indole acidic acid protein stability. Dev Cell.

[29] Reed JW, Nagpal P, Poole DS, Furuya M, Chory J (1993). Mutations in the gene for the red/far-red light receptor phytochrome B alter cell elongation and physiological responses throughout Arabidopsis development. Plant Cell.

[30] Yoshida Y, Sarmiento-Mañús R, Yamori W, Ponce MR, Micol JL, Tsukaya H (2018). The Arabidopsis phyB-9 mutant has a second-site mutation in the VENOSA4 gene that alters chloroplast size, photosynthetic traits, and leaf growth. Plant Physiol.

[31] Guo D, Song X, Yuan M, Wang Z, Ge W, Wang L, Wang J, Wang X (2017). RNA-seq profiling shows divergent gene expression patterns in Arabidopsis grown under different densities. Front Plant Sci.

[32] Ma L, Li G (2019). Auxin-dependent cell elongation during the shade avoidance response. Front Plant Sci.

[33] Procko C, Crenshaw CM, Ljung K, Noel JP, Chory J (2014). Cotyledon-generated auxin is required for shade-induced hypocotyl growth in Brassica rapa. Plant Physiol.

[34] Altangerel N, Ariunbold GO, Gorman C, Alkahtani MH, Borrego EJ, Bohlmeyer D, Hemmer P, Kolomiets MV, Yuan JS, Scully MO (2017). Reply to Dong and Zhao: plant stress via Raman spectroscopy. Proc Natl Acad Sci U S A.

[35] Huang CH, Singh GP, Park SH, Chua NH, Ram RJ, Park BS (2020). Early diagnosis and management of nitrogen deficiency in plants utilizing Raman spectroscopy. Front Plant Sci.

[36] Andreev GN, Schrader B, Schulz H, Fuchs R, Popov S, Handjieva N (2001). Non-destructive NIR-FT-Raman analyses in practice. Part 1. Analyses of plants and historic textiles. Fresenius J Anal Chem..

[37] Frosch T, Schmitt M, Noll T, Bringmann G, Schenzel K, Popp J (2007). Ultrasensitive in situ tracing of the alkaloid dioncophylline A in the tropical liana Triphyophyllum peltatum by applying deep-UV resonance Raman microscopy. Anal Chem.

[38] Dunn JL, Turnbull JD, Robinson SA (2004). Comparison of solvent regimes for the extraction of photosynthetic pigments from leaves of higher plants. Funct Plant Biol.

[39] Rustioni L, Grossi D, Brancadoro L, Failla O (2017). Characterization of iron deficiency symptoms in grapevine (Vitis Spp.) leaves by reflectance spectroscopy. Plant Physiol Biochem..

[40] Zhao YR, Li X, Yu KQ, Cheng F, He Y (2016). Hyperspectral imaging for determining pigment contents in cucumber leaves in response to angular leaf spot disease. Sci Rep.

[41] Matsuda O, Tanaka A, Fujita T, Iba K (2012). Hyperspectral imaging techniques for rapid identification of arabidopsis mutants with altered leaf pigment status. Plant Cell Physiol.

[42] Reed JW, Nagatani A, Elich TD, Fagan M, Chory J (1994). Phytochrome A and phytochrome B have overlapping but distinct functions in Arabidopsis development. Plant Physiol.

[43] Schindelin J, Arganda-Carreras I, Frise E, Kaynig V, Longair M, Pietzsch T, Preibisch S, Rueden C, Saalfeld S, Schmid B, Tinevez JY, White DJ, Hartenstein V, Eliceiri K, Tomancak P, Cardona A (2012). Fiji: an open-source platform for biological-image analysis. Nat Methods.

[44] Maloof JN, Nozue K, Mumbach MR, Palmer CM (2013). LeafJ: an ImageJ plugin for semi-automated leaf shape measurement. J Vis Exp.

[45] Lieber CA, Mahadevan-Jansen A (2003). Automated method for subtraction of fluorescence from biological Raman spectra. Appl Spectrosc.

[46] Creely CM, Singh GP, Petrov D (2005). Dual wavelength optical tweezers for confocal Raman spectroscopy. Opt Commun.

[47] Goodstein DM, Shu S, Howson R, Neupane R, Hayes RD, Fazo J, Mitros T, Dirks W, Hellsten U, Putnam N, Rokhsar DS (2012). Phytozome: a comparative platform for green plant genomics. Nucleic Acids Res..

[48] Lichtenthaler HK, Buschmann C (2001). Chlorophylls and carotenoids: measurement and characterisation by UV-VIS spectroscopy. Curr Protoc Food Analyt Chem.

