# Supplementary material for: Rapid metabolite response in leaf blade and petiole as a marker for shade avoidance syndrome
Source: Plant Methods. 2020 Oct 27;16:144. doi: 10.1186/s13007-020-00688-0 (PMC7590806; doi:10.1186/s13007-020-00688-0)
Supplement: Supplementary file 1 — Additional file 1: Fig. S1. Verification of shade conditions and determining leaf number of Arabidopsis plant for Raman spectroscopy. a Measurements of petiole length and leaf blade area of wild type (Col-0) Arabidopsis plants grown in shade conditions. Bars denote average ± SE (n = 4). b Left panel, schematic diagram of purpose-built Raman Spectroscopy system used in this study. Right panel, photograph of the Raman Spectroscopy system. Inset shows the leaf of a plant resting on the sample holder for measurement. c Left panel, Raman spectrum for each leaf number. Inset focused on 1521 cm−1 Raman peak. Right panel, measured peak intensities at 1521 cm−1 Raman shift. Bars denote average ± SE (n = 8). d Development of leaf number 3 of Arabidopsis plant. Numbers represent leaf number, according to order of development. C represents cotyledon. Scale, 1 cm. WL, white light; MS, moderate shade; DS, deep shade; DAG, days after germination. Fig. S2. Raman spectra of Arabidopsis plant under shade conditions. a Raman spectra of Arabidopsis leaf blades and petioles under different shade conditions. (leaf blade: n = 8, petiole: n = 4). b Three-dimensional principal component analysis (PCA) plot of wild type (Col-0) Arabidopsis under shade conditions. c Raman spectra for carotenoid standards. WL, white light; MS, moderate shade; DS, deep shade. Fig. S3. Raman spectra of same-age wild type (Col-0) Arabidopsis plants with different duration of shade treatment. Number of hours (h) or days (d) represents the duration of shade treatment. Control plants are not exposed to shade. (leaf blade: n = 10, petiole: n = 8). Fig. S4. Raman spectra of Arabidopsis plants from seedling to mature stage in time-course shade experiment. a Phenotype of plants at different age and different durations of shade treatment. 10-days-old plants were subjected to 7 days treatment of white light (WL) or deep shade (DS). b Carotenoids Raman peak of plants in a. Dashed line indicates difference in peak intensity [file 13007_2020_688_MOESM1_ESM.pdf]

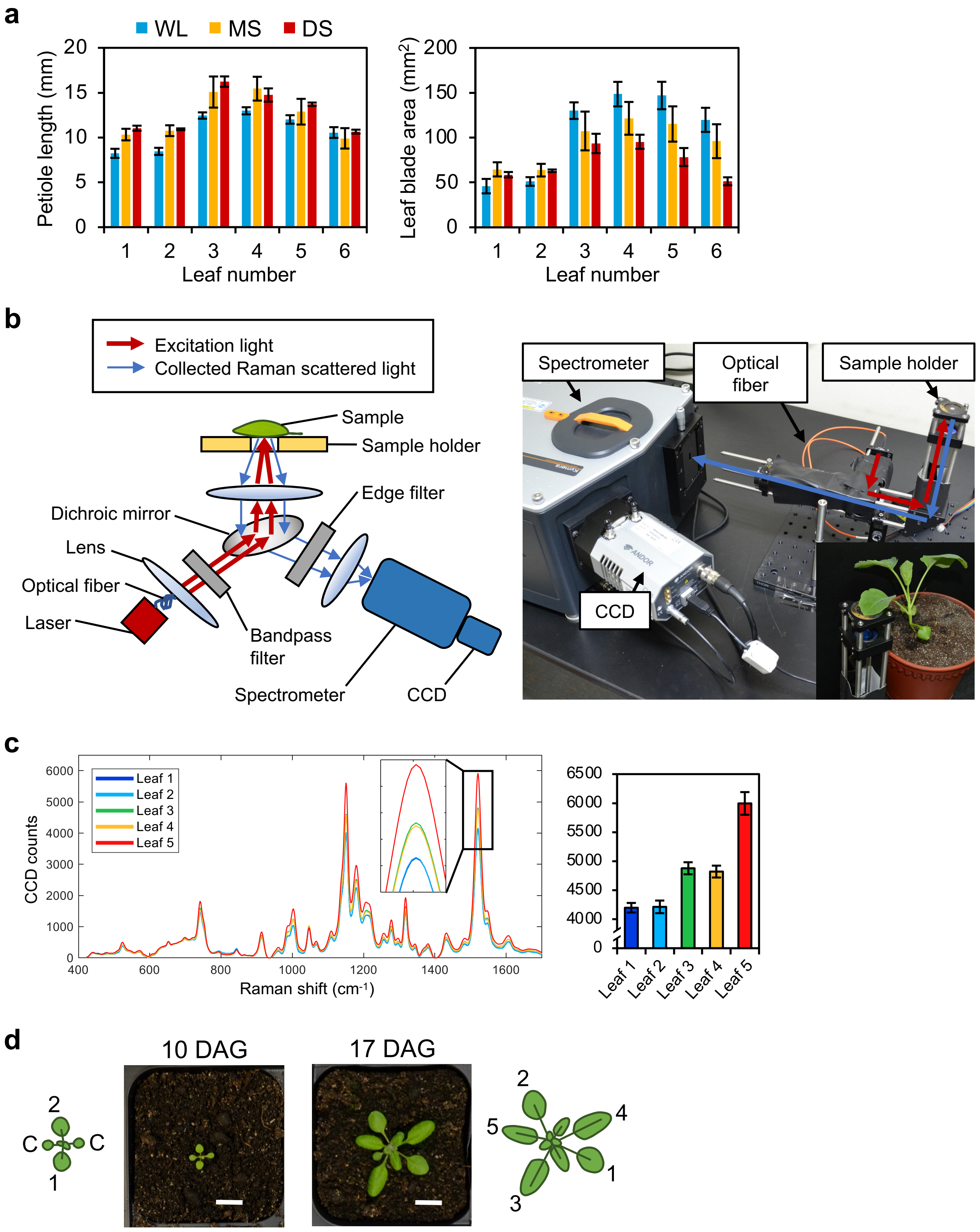

Fig. S1 Verification of shade conditions and determining leaf number of Arabidopsis plant for Raman spectroscopy. **a** Measurements of petiole length and leaf blade area of wild type (Col-0) Arabidopsis plants grown in shade conditions. Bars denote average ± SE (n=4). **b** Left panel, schematic diagram of purpose-built Raman Spectroscopy system used in this study. Right panel, photograph of the Raman Spectroscopy system. Inset shows the leaf of a plant resting on the sample holder for measurement. **c** Left panel, Raman spectrum for each leaf number. Inset focused on 1521 cm^{-1} Raman peak. Right panel, measured peak intensities at 1521 cm^{-1} Raman shift. Bars denote average ± SE (n=8). **d** Development of leaf number 3 of Arabidopsis plant. Numbers represent leaf number, according to order of development. C represents cotyledon. Scale, 1cm. WL, white light; MS, moderate shade; DS, deep shade; DAG, days after germination.

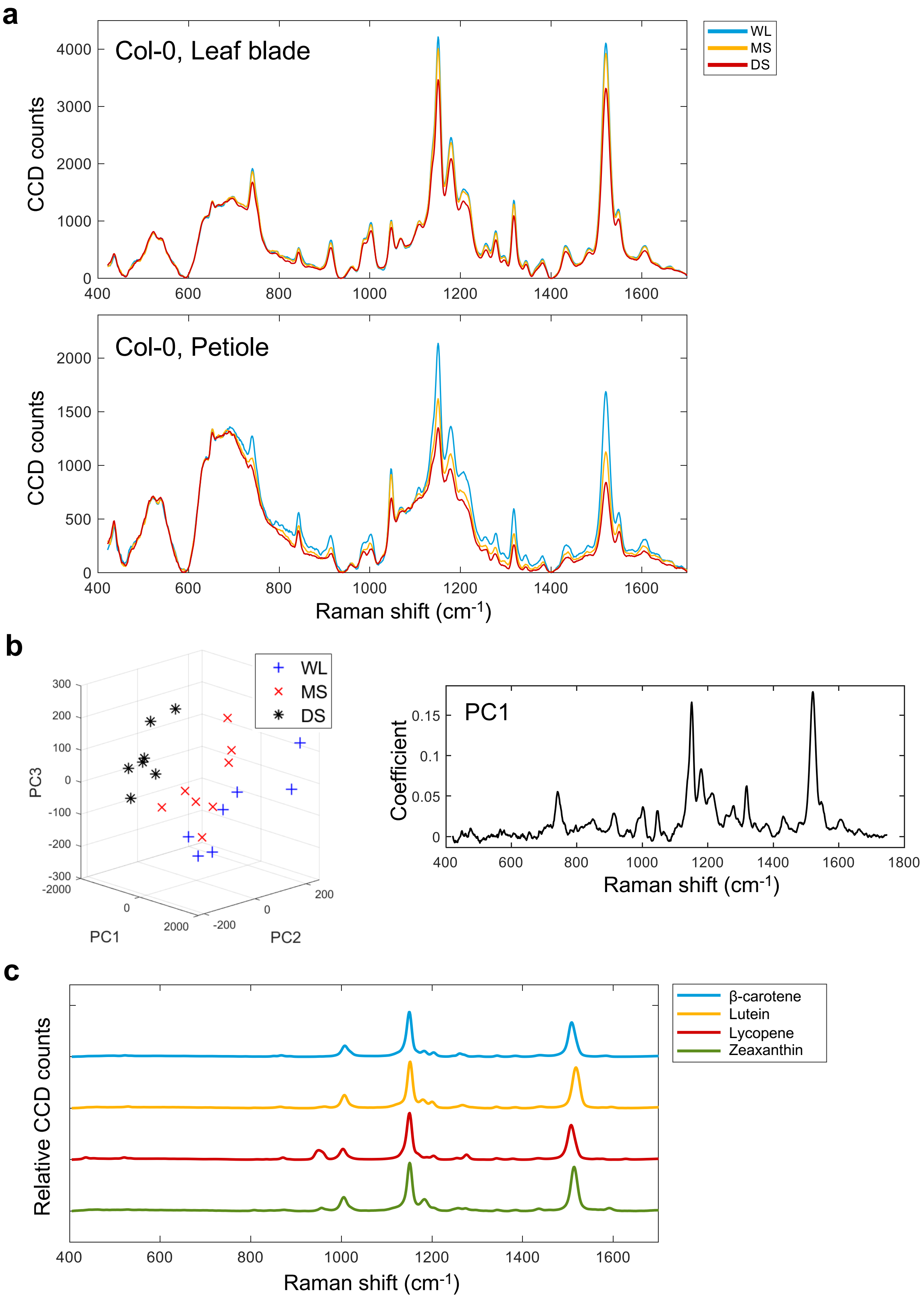

Fig. S2 Raman spectra of Arabidopsis plant under shade conditions. **a** Raman spectra of Arabidopsis leaf blades and petioles under different shade conditions. (leaf blade: n=8, petiole: n=4). **b** Three-dimensional principal component analysis (PCA) plot of wild type (Col-0) Arabidopsis under shade conditions. **c** Raman spectra for carotenoid standards. WL, white light; MS, moderate shade; DS, deep shade.

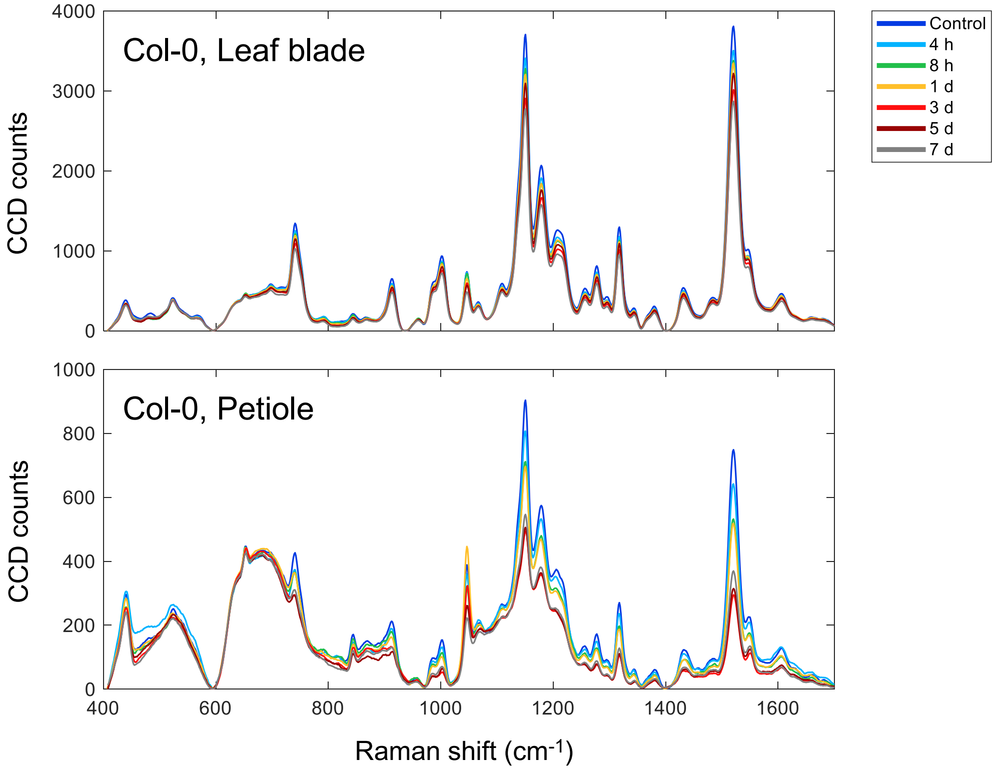

Fig. S3 Raman spectra of same-age wild type (Col-0) Arabidopsis plants with different duration of shade treatment. Number of hours (h) or days (d) represents the duration of shade treatment. Control plants are not exposed to shade. (leaf blade: n=10, petiole: n=8).

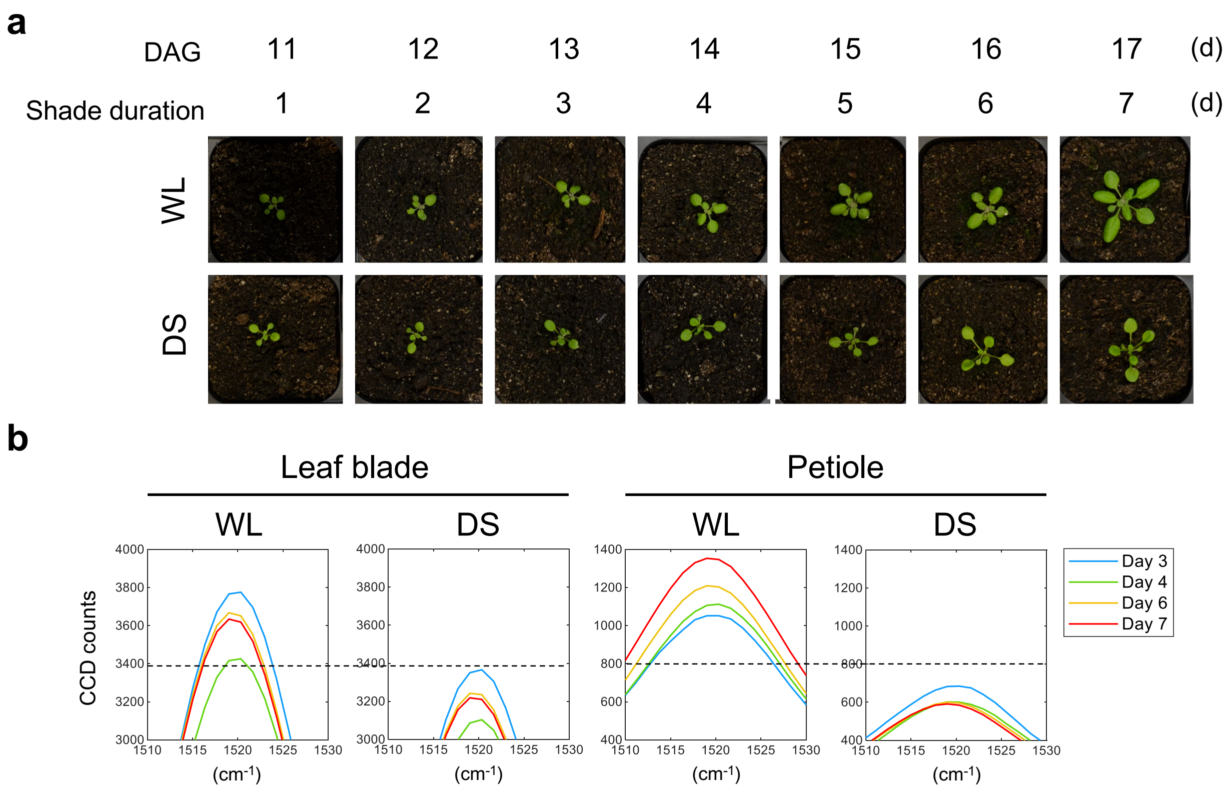

Fig. S4 Raman spectra of Arabidopsis plants from seedling to mature stage in time-course shade experiment. **a** Phenotype of plants at different age and different durations of shade treatment. 10 d-old plants were subjected to 7 d treatment of white light (WL) or deep shade (DS). **b** Carotenoids Raman peak of plants in **a**. Dashed line indicates difference in peak intensity between WL and DS. DAG, days after germination.

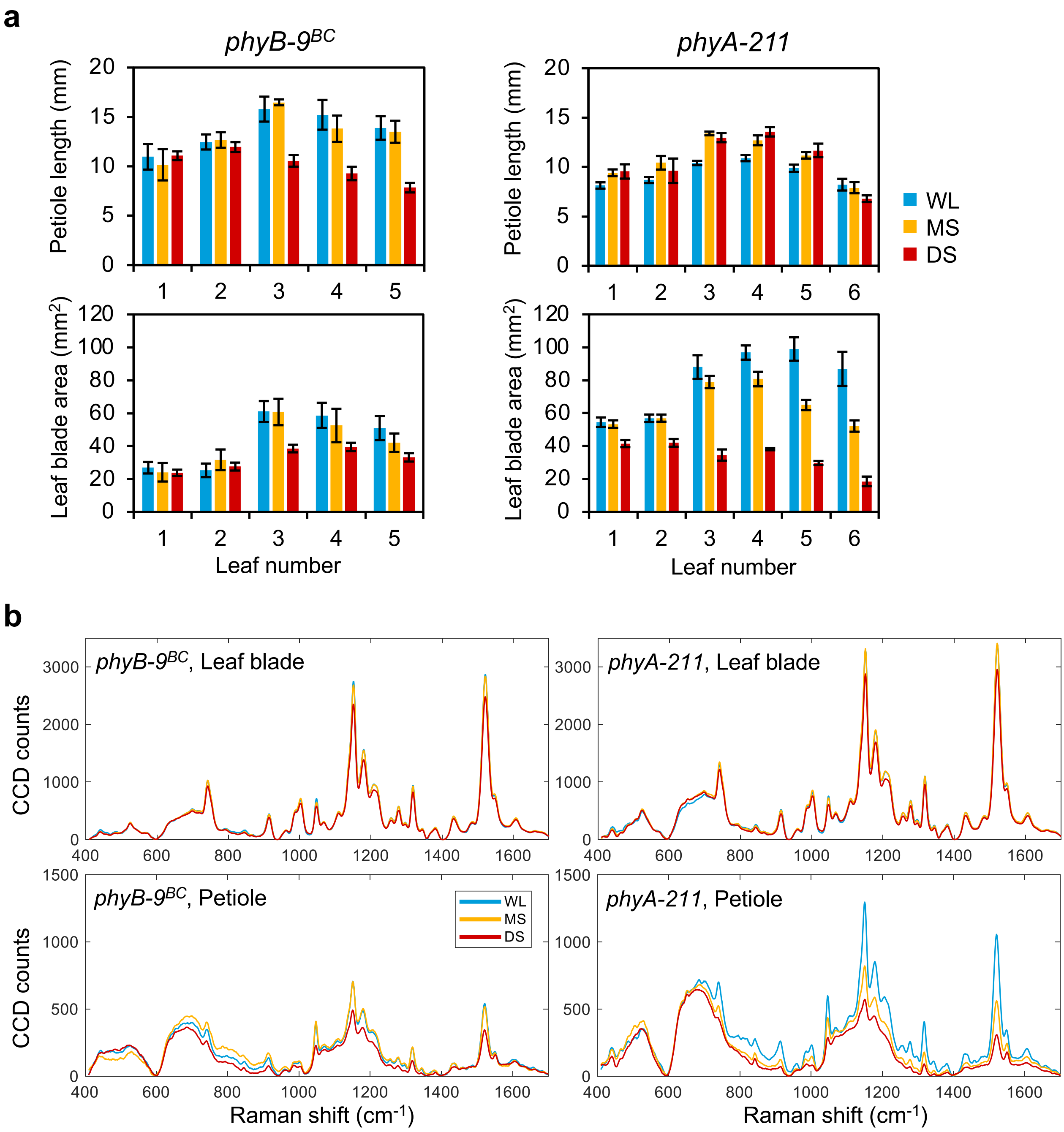

Fig. S5 Raman spectra of Arabidopsis phytochrome mutants under shade conditions. **a** Petiole length and leaf blade area of *phyB-9*^{BC} and *phyA-211* under shade. Bars denote average ± SE (n=3). **b** Raman spectra of leaf blades and petioles of *phyB-9*^{BC} and *phyA-211* in **a** (leaf blade: n=8, petiole: n=4). WL, white light; MS, moderate shade; DS, deep shade.

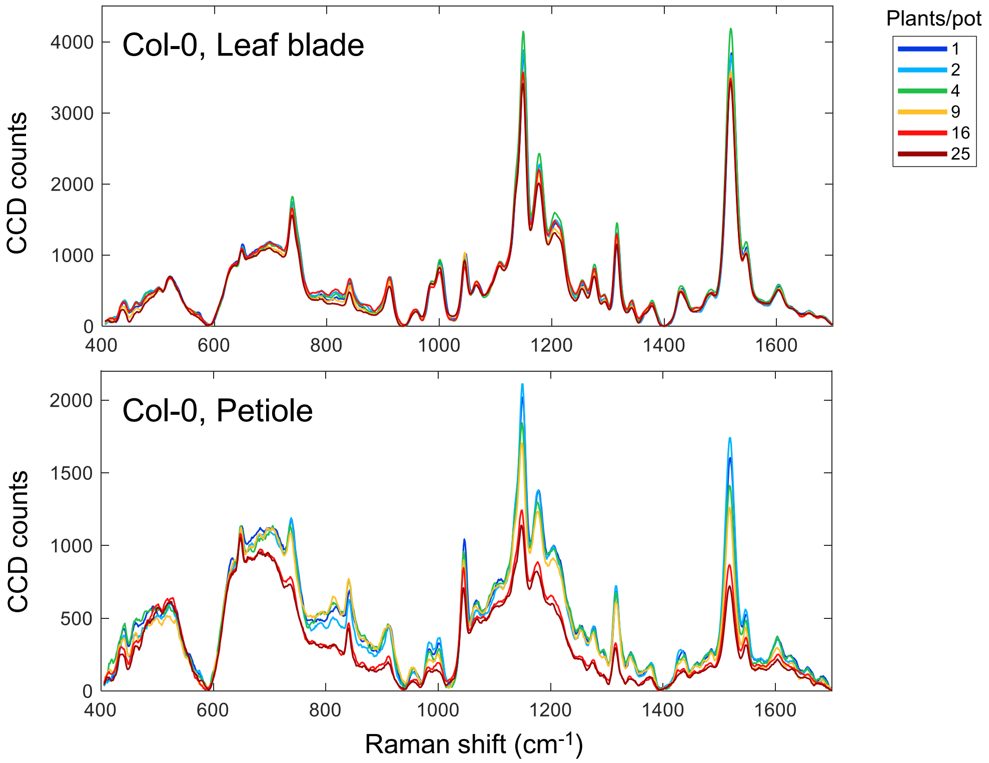

Fig. S6 Raman spectra of wild type (Col-0) Arabidopsis leaf blades and petioles in low to high density planting. (leaf blade: n=5, petiole: n=3).

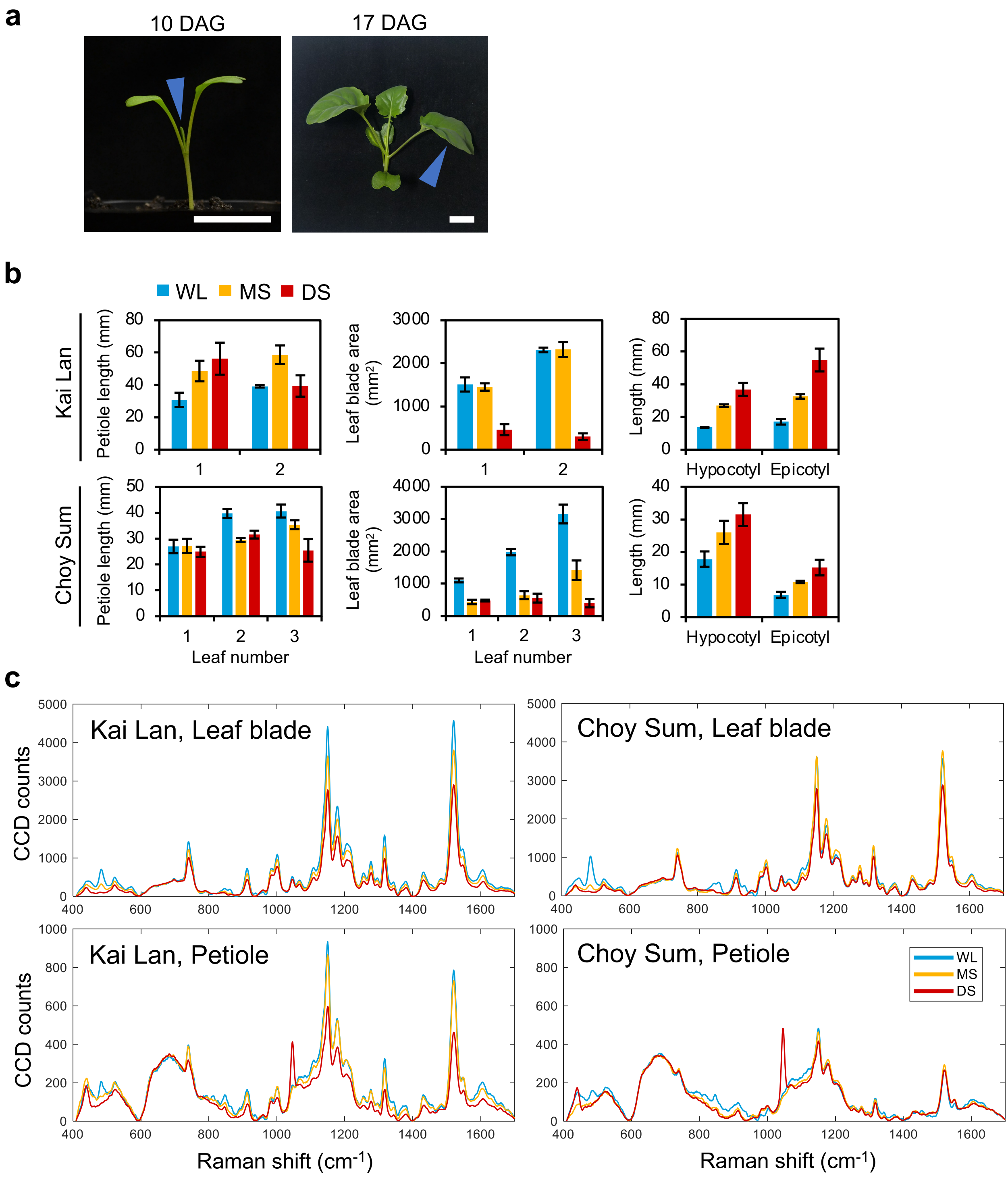

Fig. S7 Raman spectra of leafy vegetables under shade conditions. **a** Development of leaf number 1 (blue arrowhead) in vegetables. Scale, 2 cm. **b** Measurements of petiole length, leaf blade area, hypocotyl length, and epicotyl length of Kai Lan and Choy Sum grown in shade conditions. Bars denote average ± SE (n=3). **c** Raman spectra of leaf blades and petioles of Kai Lan and Choy Sum in **a** (leaf blade: n=8, petiole: n=4). WL, white light; MS, moderate shade; DS, deep shade; C, cotyledon.

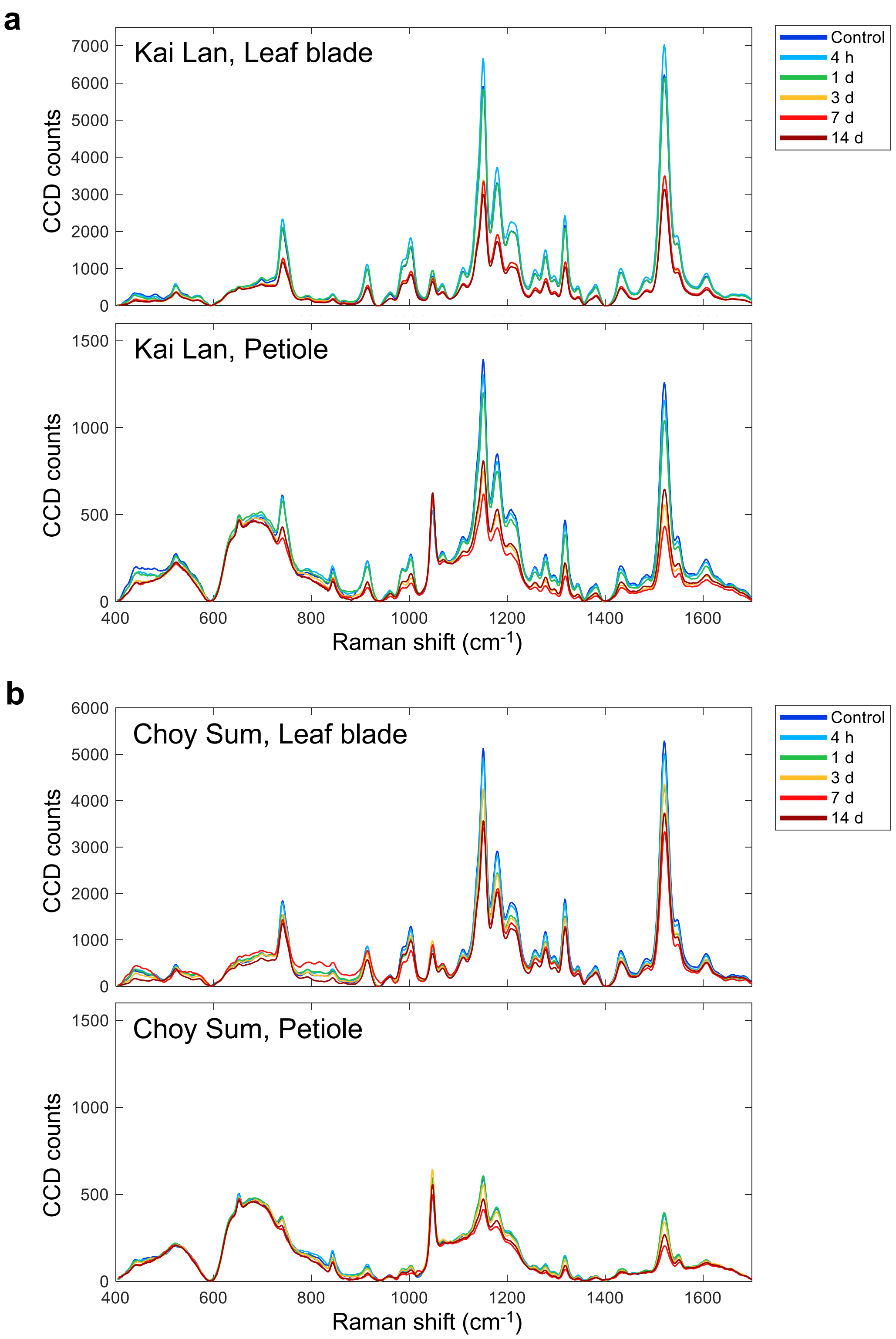

Fig. S8 Raman spectra of leafy vegetables with different duration of shade treatment. **a** Raman spectra of leaf blades and petioles of Kai Lan (n=5). **b** Raman spectra of leaf blades and petioles of Choy Sum (n=4).

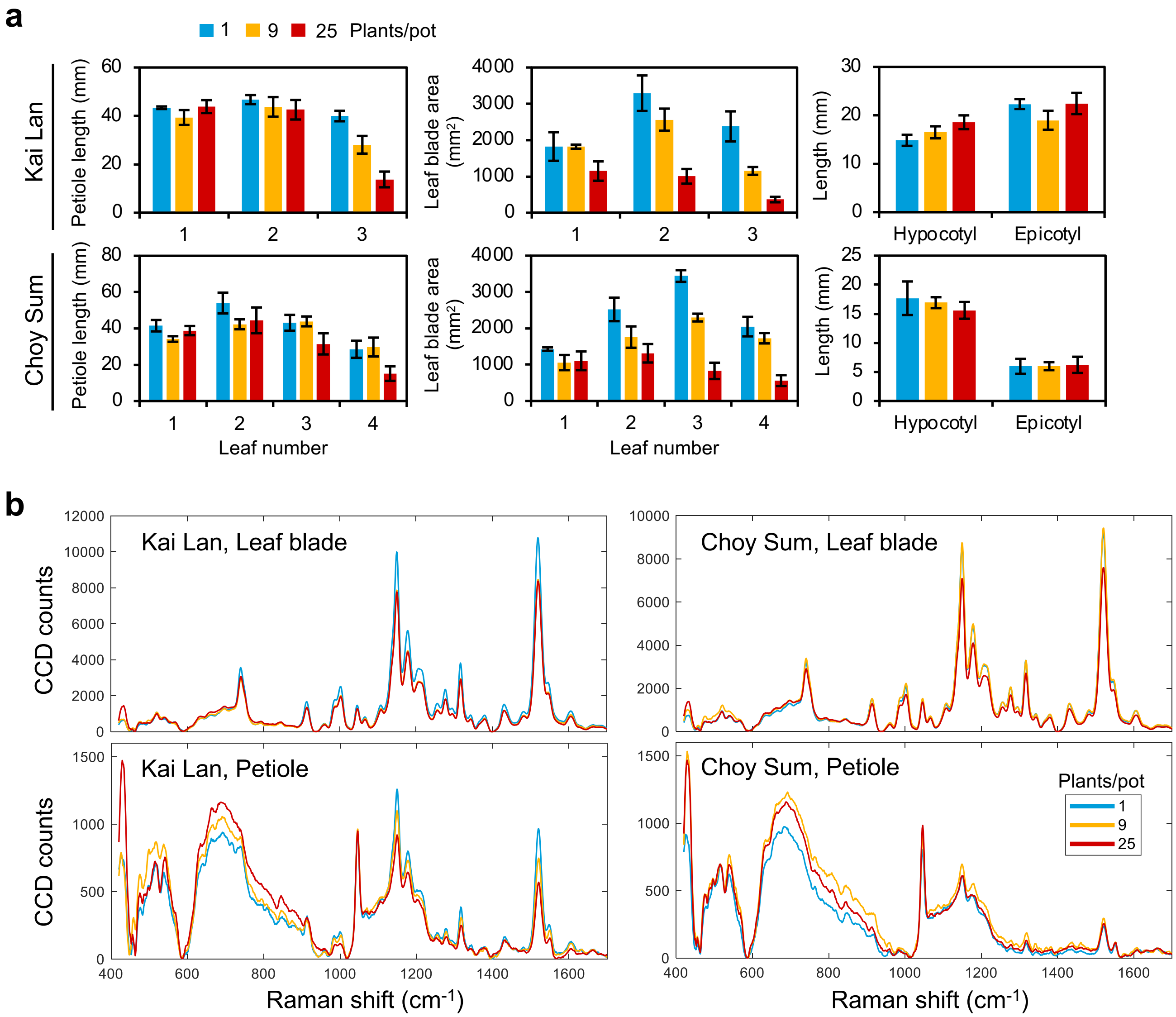

Fig. S9 Raman spectra of leafy vegetables in high density planting. **a** Measurements of petiole length, leaf blade area, hypocotyl length, and epicotyl length of Kai Lan and Choy Sum grown in low to high density conditions. Bars denote average ± SE (n=4). **b** Raman spectra of leaf blades and petioles of Kai Lan and Choy Sum in **a** (leaf blade: n=6, petiole: n=4).

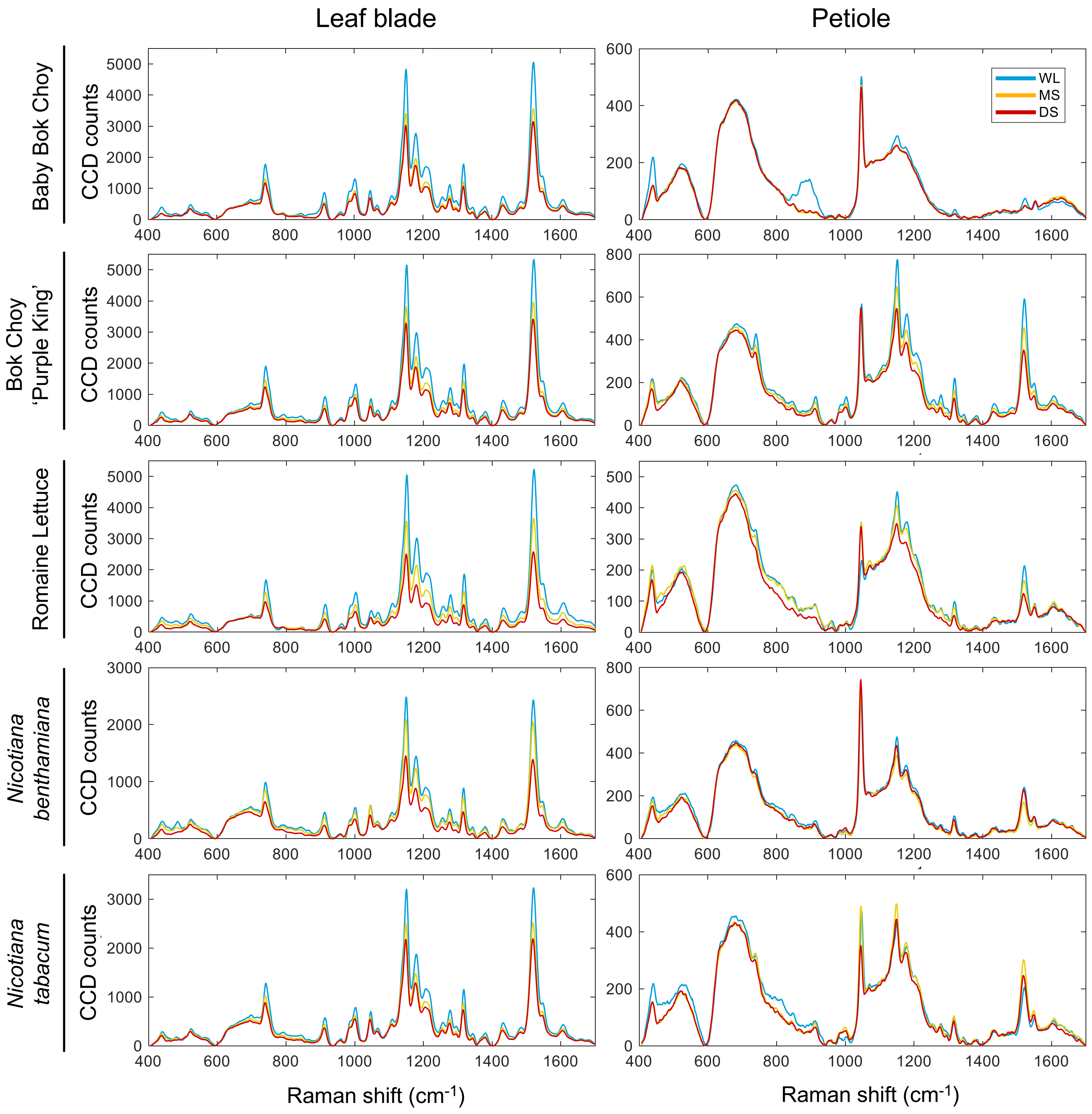

Fig. S10 Raman spectra of various plant species under shade conditions. (Baby Bok Choy: leaf blade n=7, petiole n=4; Bok Choy 'Purple King': leaf blade n=6, petiole n=6; Romaine Lettuce: leaf blade n=6, petiole n=6; *Nicotiana benthamiana*: leaf blade n=3, petiole n=3; *Nicotiana tabacum*: leaf blade n=6, petiole n=3). WL, white light; MS, moderate shade; DS, deep shade.
