# Supplementary figures and images for: Rapid metabolite response in leaf blade and petiole as a marker for shade avoidance syndrome
Source: Plant Methods. 2020 Oct 27;16:144. doi: 10.1186/s13007-020-00688-0 (PMC7590806; doi:10.1186/s13007-020-00688-0)

**Table S1. List of primers used in qRT-PCR analysis.**


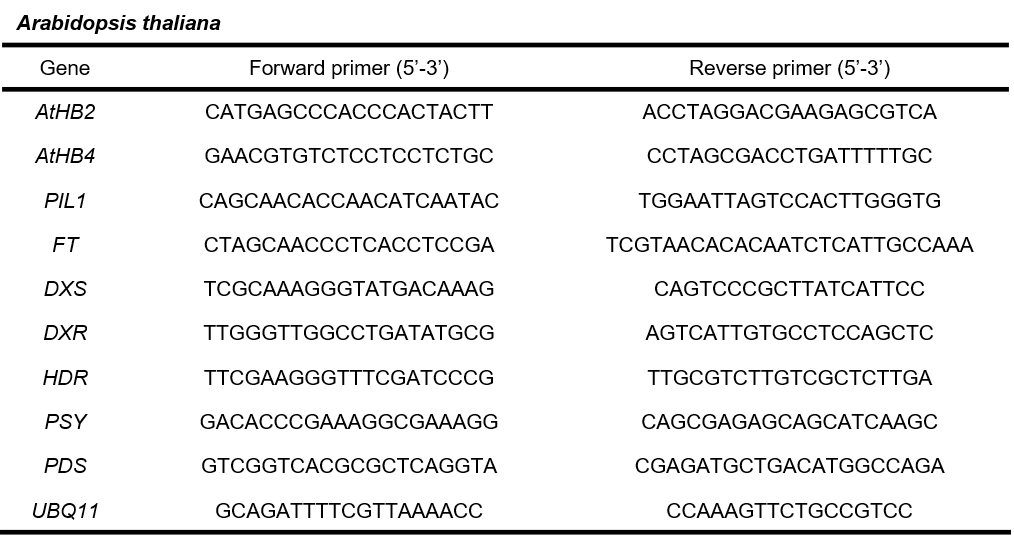

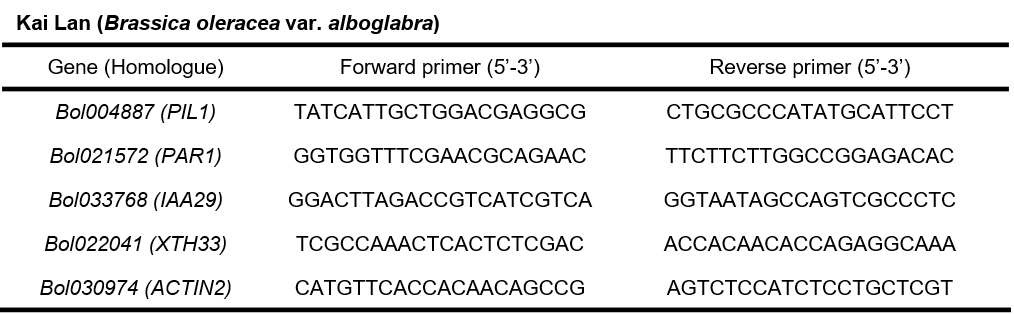

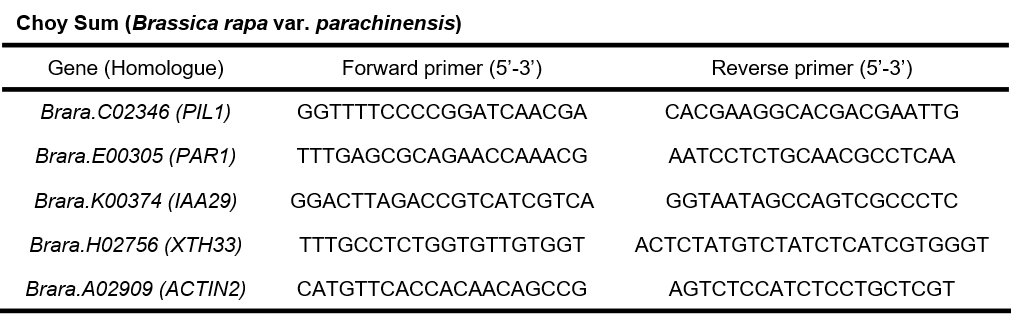

Supplement: Supplementary file 2 — Additional file 2: Table S1. List of primers used in qRT-PCR analysis. [file 13007_2020_688_MOESM2_ESM.docx]
